# Versatile Dynamic Motion Generation Framework: Demonstration With a Crutch-Less Exoskeleton on Real-Life Obstacles at the Cybathlon 2020 With a Complete Paraplegic Person

**DOI:** 10.3389/frobt.2021.723780

**Published:** 2021-09-24

**Authors:** Vaiyee Huynh, Guillaume Burger, Quoc Viet Dang, Raphaël Pelgé, Guilhem Boéris, Jessy W. Grizzle, Aaron D. Ames, Matthieu Masselin

**Affiliations:** ^1^ Wandercraft Company, Paris, France; ^2^ Department of Electrical Engineering and Computer Science, University of Michigan, Ann Arbor, MI, United States; ^3^ Department of Mechanical and Civil Engineering, California Institute of Technology, Pasadena, CA, United States

**Keywords:** lower-limb exoskeleton, self-balanced, optimal control, direct collocation, trajectory generation, cybathlon

## Abstract

Lower-limb exoskeletons are a promising option to increase the mobility of persons with leg impairments in a near future. However, it is still challenging for them to ensure the necessary stability and agility to face obstacles, particularly the variety that makes the urban environment. That is why most of the lower-limb exoskeletons must be used with crutches: the stability and agility features are deferred to the patient. Clinical experience shows that the use of crutches not only leads to shoulder pain and exhaustion, but also fully occupies the hands for daily tasks. In November 2020, Wandercraft presented Atalante Evolution, the first self-stabilized and crutch-less exoskeleton, to the powered exoskeleton race of the Cybathlon 2020 Global Edition. The Cybathlon aims at promoting research and development in the field of powered assistive technology to the public, contrary to the Paralympics where only participants with unpowered assistive technology are allowed. The race is designed to represent the challenges that a person could face every day in their environment: climbing stairs, walking through rough terrain, or descending ramps. Atalante Evolution is a 12 degree-of-freedom exoskeleton capable of moving dynamically with a complete paraplegic person. The challenge of this competition is to generate and execute new dynamic motions in a short time, to achieve different tasks. In this paper, an overview of Atalante Evolution system and of our framework for dynamic trajectory generation based on the direct collocation method will be presented. Next, the flexibility and efficiency of the dynamic motion generation framework are demonstrated by our tools developed for generating the important variety of stable motions required by the competition. A smartphone application has been developed to allow the pilot to choose between different modes and to control the motion direction according to the real situation to reach a destination. The advanced mechatronic design and the active cooperation of the pilot with the device will also be highlighted. As a result, Atalante Evolution allowed the pilot to complete four out of six obstacles, without crutches. Our developments lead to stable dynamic movements of the exoskeleton, hands-free walking, more natural stand-up and turning moves, and consequently a better physical condition of the pilot after the race compared to the challengers. The versatility and good results of these developments give hope that exoskeletons will soon be able to evolve in challenging everyday-life environments, allowing patients to live a normal life in complete autonomy.

## 1 Introduction

The Cybathlon 2020 Global Edition is a championship that gathered fifty-one teams from all over the world in six disciplines: powered arm prosthesis, brain-computer interface, powered exoskeleton, functional electrical stimulation bike (PNF et al. (2020)), powered leg prosthesis and powered wheelchair (official website: https://cybathlon.ethz.ch/). The aim of the Cybathlon is to promote research and development in the field of powered assistive technology to the public (Erdmann et al. (2020)). Since the first edition in 2016 (Erdmann et al. (2020)), the Cybathlon organization has worked hard to define the obstacles of the races representing everyday challenges for people with disabilities and to set up a fair competition.

The use of lower-limb exoskeletons is not common because they are not yet adapted to face the complex environment that surrounds us, but they promise a real difference in comparison to wheelchairs. For people who spend their life seated, exoskeletons could improve their physical and psychological health. Most of the exoskeletons for paraplegic persons or for rehabilitation purposes (Chen et al. (2016); Kapsalyamov et al. (2019); Kalita et al. (2020)) require external support mechanisms such as crutches or canes, and only a few of them are self-balanced (e.g., REX (Contreras-Vidal and Grossman (2013))). Having an exoskeleton without crutches is a real challenge because the robot is mainly responsible for the performances of the walk such as the balance and the speed. Some hardware compromises need to be made for instance between the weight of the machine, the stability control and motion requirements (Rupal et al. (2017)). Wandercraft decided to design and develop crutch-less exoskeletons, such as Atalante, to provide autonomy as much as possible to its wearer. Atalante is CE marked and offers a stable bipedal walk and dynamic motions to the patient whose weight can be up to 90 kg (Agrawal et al. (2017); Harib et al. (2018); Gurriet et al. (2018)). We adapted and improved Atalante software and interfaces to compete in the powered exoskeleton race: this gives birth to Atalante Evolution the robot we presented at the Cybathlon championship, with Kevin Piette as pilot.

**FIGURE 1:**
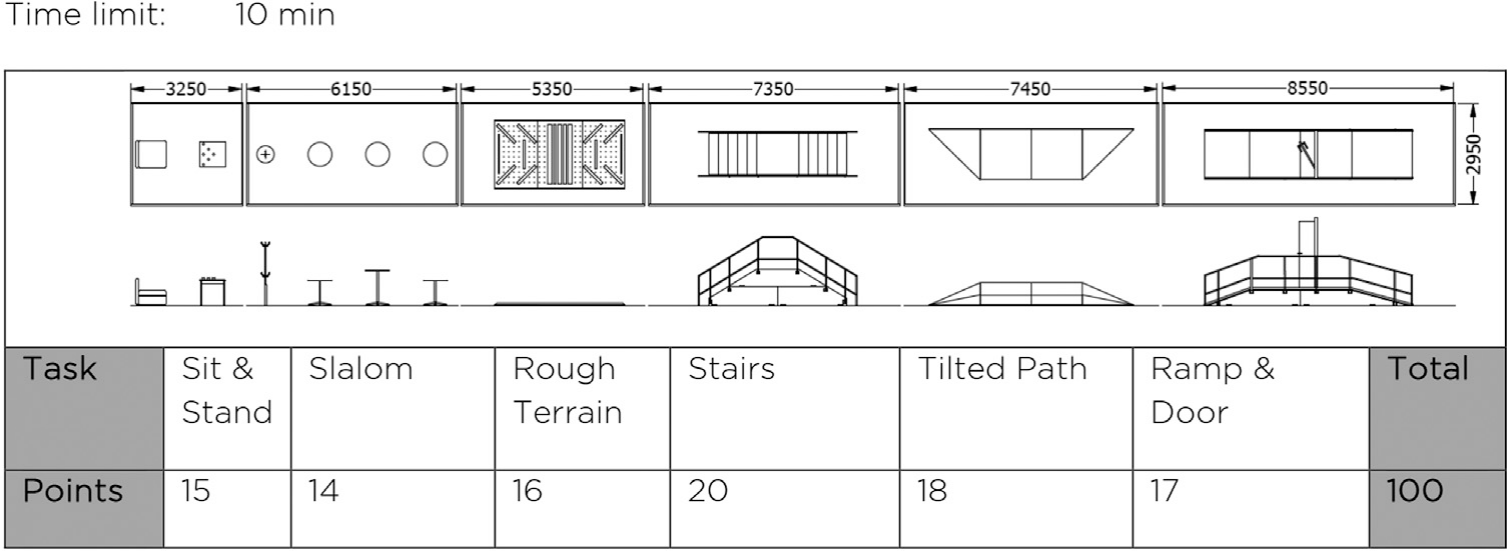
EXO race track from Cybathlon’s rules.

The powered exoskeleton race designed by the Cybathlon team is composed of six tasks (Figure 1): 1- Sit and Stand task, stand up from a sofa and stack cups on a table; 2- Slalom task, slaloming between tables; 3- Rough Terrain task, cross an uneven terrain; 4- Stairs task, going up and down six steps; 5- Tilted path task, cross a tilted terrain of 16° with synthetic grass; 6- Ramp and Door task, ascend a 20° slope, open and close a door, and descend a 15° slope. Every task scores a number of points in function of how hard the obstacle is, and the winner is the fastest team with the highest score. Of all competing exoskeletons presented in this race, only Wandercraft’s Atalante Evolution presented without crutches.

**FIGURE 2:**
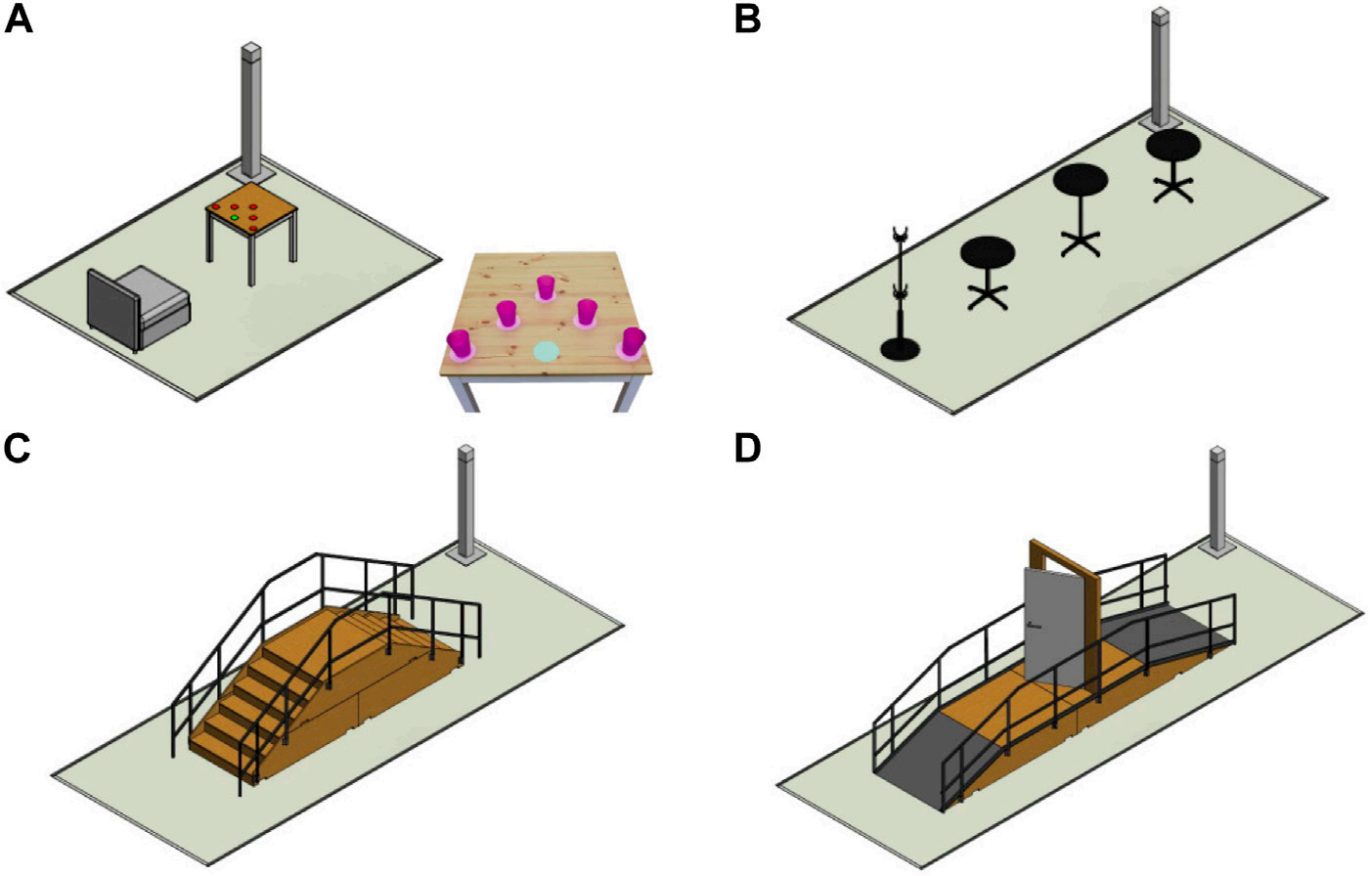
**(A)** Sit and Stand task; **(B)** Slalom task; **(C)** Stairs task; **(D)** Ramp and Door.

This is the first time that a crutch-less exoskeleton enters the EXO race and tries to go through the obstacles. For the 2020’s edition, we decided to focus on four tasks over the six (Figure 2): Sit and Stand task, Slalom task, Stairs task and Ramp and Door task. Two reasons motivated this decision. First, the timings were short so we decided to focus our resources on a subset. Second, our hardware made some tasks very challenging. The ankle amplitude in the frontal plane was not sufficient to walk on the tilted plane while keeping the feet flat on the surface. Likewise, the sole of the feet was not appropriate to walk on an uneven terrain while using the force sensors.

Analyzing the chosen tasks, we decomposed them in essential but challenging motions such as:

1) Position oneself precisely in front of obstacles: multi-directional steps.
2) Stand up from a soft and low seat.
3) Changing walking direction quickly, in narrow spaces while keeping stability.
4) Going up and down with limited ankle joints range and soles longer than the steps of the staircase.
5) Going up a 20° slope and going down a 15° slope with limited ankle joints range.

To achieve these motions, new features were implemented and a new user interface was developed to improve the usability of the device in a short time. Thus, releasing a new feature implies:

1) generate new dynamic motion trajectories using a Direct Collocation algorithm (section 3),
2) execute them on Atalante Evolution (section 4),
3) iterate with the pilot to improve the stability and the fluidity of the motion (subsection 4.2).

“Ordinary life for extraordinary people”. This is the leitmotiv of Wandercraft. We believe that freeing the hands is important for a lower-limb exoskeleton: having gestures or manipulating things while standing or walking should be possible for everyone. The environment around us is highly non-standardized and the exoskeleton should be the vehicle that takes people with leg impairments out. In this context, it is extremely important to be able to address very quickly any new environment. In this paper, we begin with an overview of Atalante Evolution hardware, the control architecture and the smartphone application as user interface, for mode selection and movement direction control. Our framework for dynamic motion generation based on the direct collocation method will be then presented. Next, the flexibility and efficiency of this framework are proved by our tools developed for generating the important variety of stable motions required by the competition. By “efficiency”, we mean our capacity to handcraft and adjust new trajectories quickly for a user. We described the results we obtained for the new features and how we succeed to make them stable and comfortable jointly with the pilot. We conclude with the importance to continue improving stability and dynamic motions for Atalante and for any future crutch-less exoskeletons that allow a person to face any obstacle of the urban environment in complete autonomy.

## 2 System Overview

### 2.1 Atalante Evolution Exoskeleton

Atalante, in reference to the Greek heroine of running, has been developed and improved all along the company life and the current version is the fifth iteration. For its nominal use in rehabilitation centers, a winch is required to ensure the security of the patient and a physiotherapist shall assist them, for instance by helping them to install in the exoskeleton. Atalante Evolution is outside the medical context and was developed as a prototype with improved features. The security of the pilot remains our priority, that is why four spotters are required around them during the race to replace the use of a winch. In the rules of the race, if any spotter touches the pilot during the performance, the task is considered failed.

**FIGURE 3:**
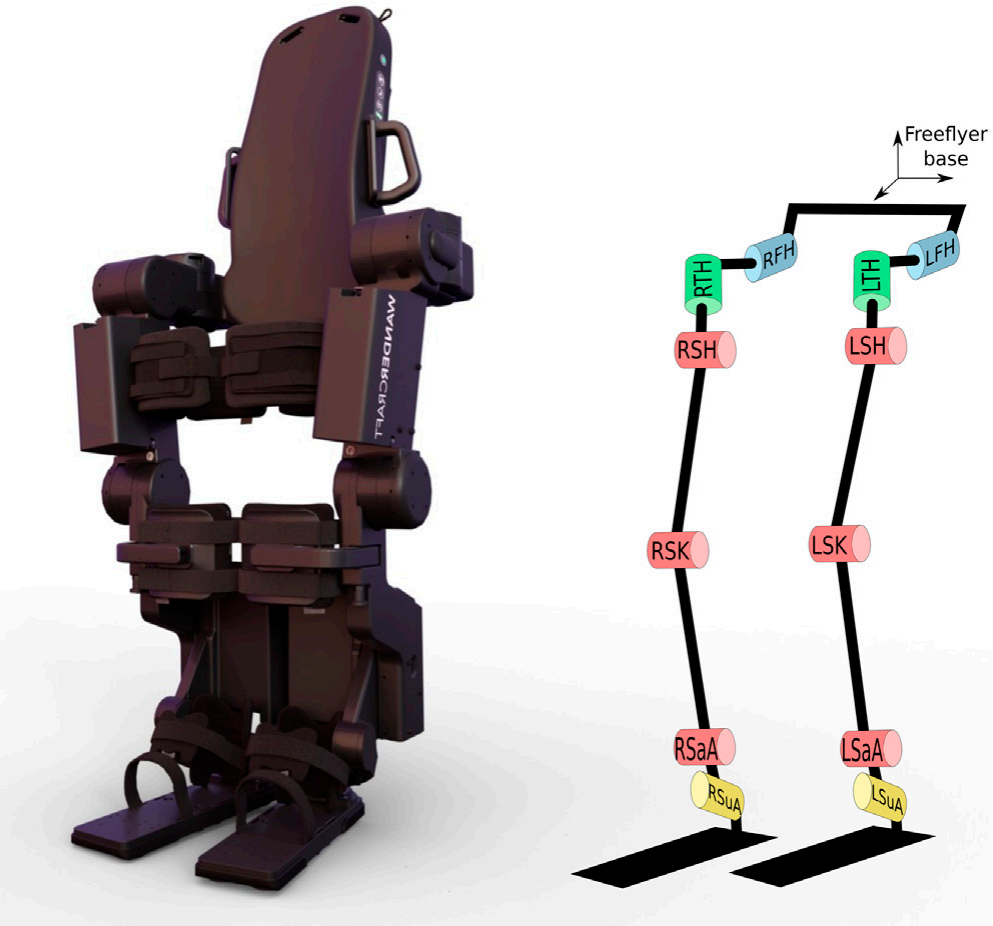
3D rendering **(left)** and kinematic tree **(right)** of the Atalante exoskeleton.

**FIGURE 4:**
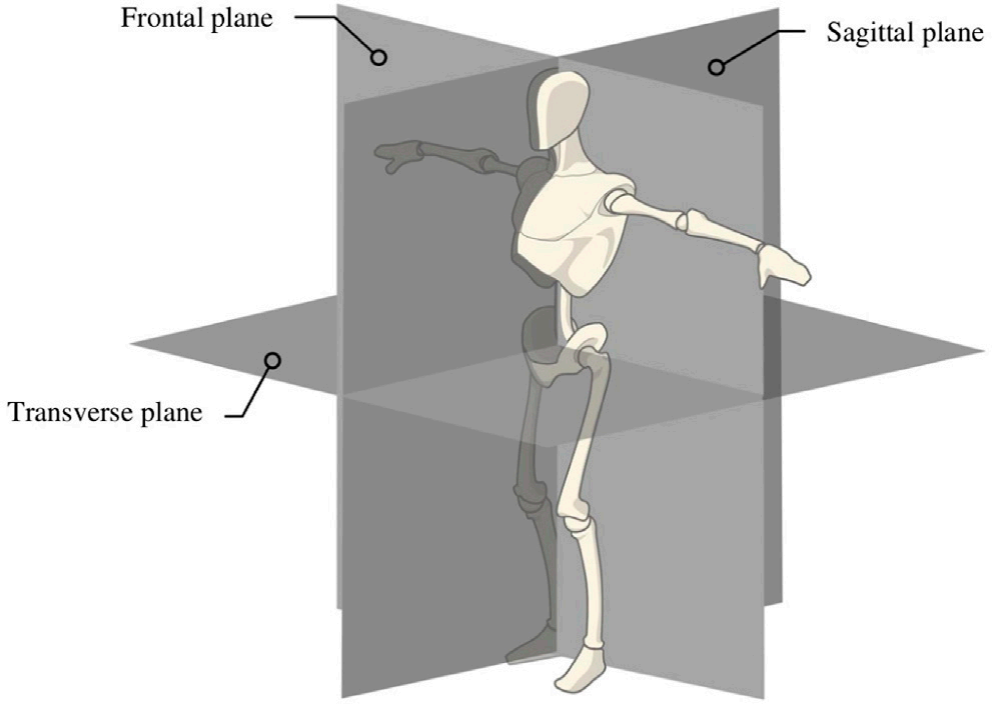
Definition of the subtalar axis.

**FIGURE 5:**
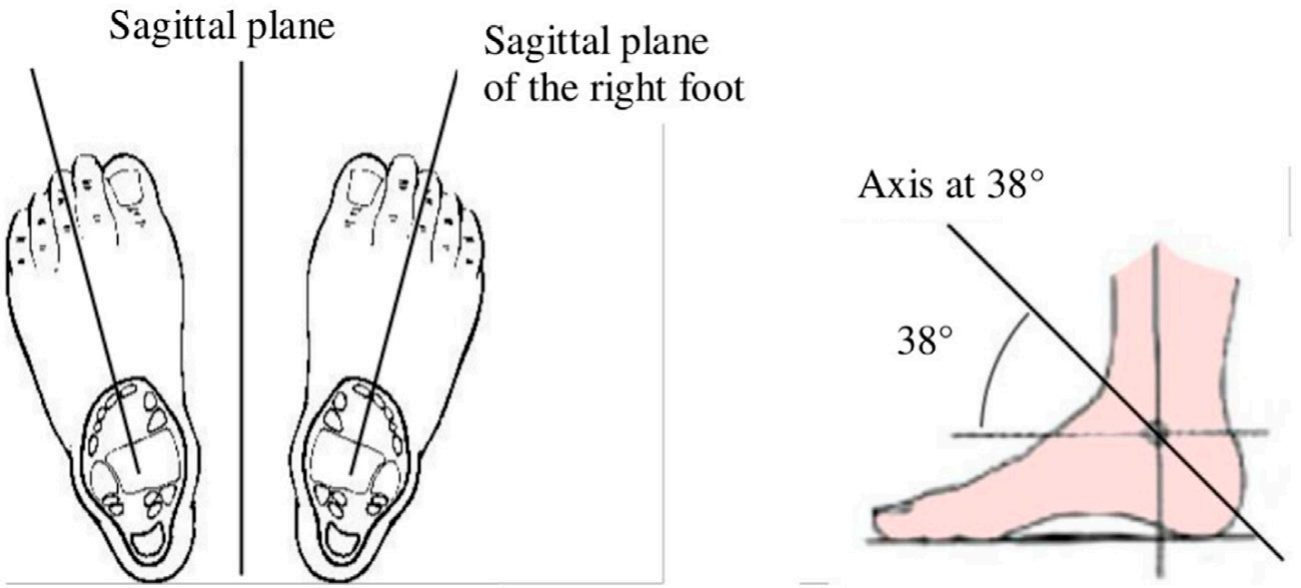
Definition of the subtalar axis.

The robot weighs 82 kg and it has 12 degrees of freedom (Figure 3), fully actuated by 12 electric motors. It can reproduce the human gait in an anthropomorphic way: three actuators at each hip reproducing the human motion around sagittal, transverse and frontal axes (Figure 4), one actuator at each knee around the sagittal knee axis and two actuators at each ankle around sagittal and subtalar axes (Lux (2018)) (Figure 5). On the hips and the knees, each motor is directly coupled to one reducer to actuate one axis. The reduction is made with Harmonic Drive gearboxes. The ankle transmission is a parallel mechanism: two motors are necessary to actuate jointly two ankle axes. The power transmission is made through a screw balls and rods on each motor output and the reduction factor is variable and needs to be computed for each position. Two 48 V batteries power them. It is equipped with electronic boards and sensors to control and compute the motions. The displacement and velocity of each actuated joint are measured by a digital encoder mounted on the corresponding motor. Four low-cost Inertial Measurement Units (IMU) are mounted in the exoskeleton: one attached to the patient torso on the jacket, one in the exoskeleton’s pelvis and one in each leg of the exoskeleton above the ankles. It has been shown in Vigne et al. (2020) that these IMU were enough to estimate correctly the mechanical deformations. In addition, under each sole, four 1-axis force sensors are mounted to detect ground contact.

To fit the patient’s lower limbs, the exoskeleton’s legs are manually adjustable. The exoskeleton has seven body interfaces to fasten the user:

• three fasteners with straps per leg (thigh, leg, and foot), designed to avoid high-pressure areas and that can rotate to adapt to each morphology,
• a jacket with hip foams to maintain the user’s upper body to the exoskeleton’s back.

### 2.2 Control System Architecture Overview

**FIGURE 6:**
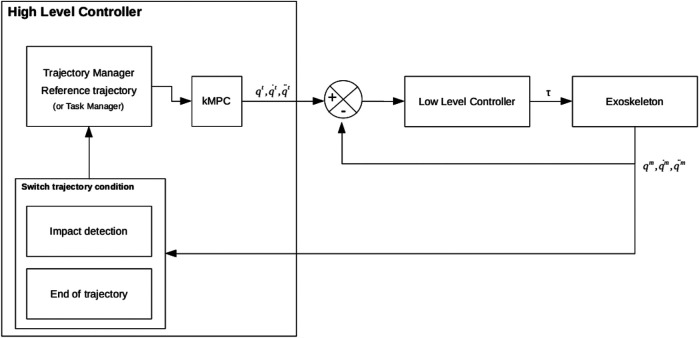
Atalante Evolution’s control architecture overview schema.

The control system architecture is mainly composed of three components: the High Level Controller, the Low Level Controller and the robot system (Figure 6).

The High Level Controller (HLC) computes the target joint positions **q** ^*t*^, velocities ${\dot{q}}^{t}$ and accelerations ${\ddot{q}}^{t}$ to give to the Low Level Controller (LLC). The targets could come from a task manager with an inverse kinematics algorithm or from a trajectory manager with a controller that executes the trajectories. In this paper, we focus on the trajectory part only. One trajectory corresponds to one motion with a specific contact configuration (see Section 3). The trajectory is considered as finished when an impact is detected through the force sensors or when the whole trajectory is executed. In case of walking or stepping and to absorb the shock of the impact on the floor, the ankles are compliant during the impact period before tracking the reference trajectory again. A kinematic Model Predictive Controller (kMPC) is used to ensure the continuity of the targets and smooths the output trajectory. The associated cost function minimizes the joint position, velocity, acceleration, and jerk errors while respecting boundary constraints.

The LLC is the layer that controls all the joints. Each joint is position controlled and has a proportional-derivative-integrator controller that converts the errors between target joint positions, velocities and the measured ones (⋅^*m*^) to torques for the motors of the robot system following this equation:

$$\tau =K_{p}\left(e+K_{d}\dot{e}+K_{i}\int e\right)$$ (1)

with

• *τ*: joint torque
• *K*_*p*_, *K*_*d*_ and *K*_*i*_: proportional, derivative and integrative gains respectively
• *e *: joint position error

The HLC runs at 200 Hz whereas the LLC and the sensor feedback from the physical system run at 1 kHz.

### 2.3 A Smartphone Application: CybAGUI

**FIGURE 7:**
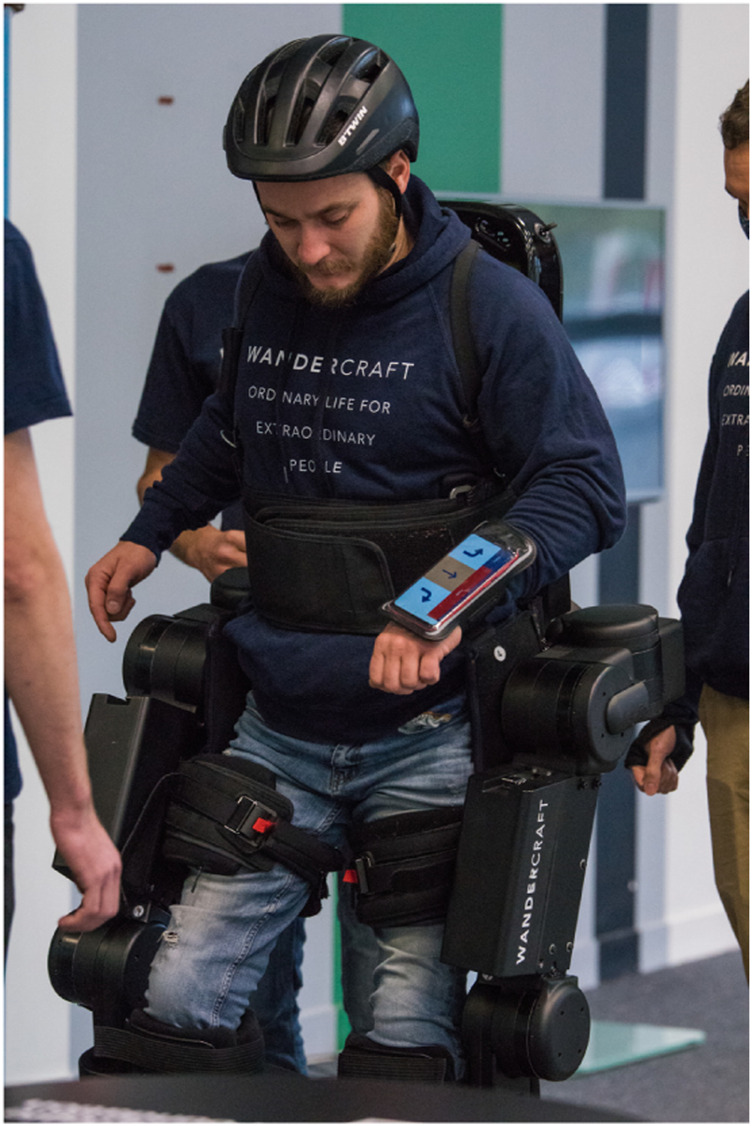
Kevin Piette at EXO race of Cybathlon Global Edition 2020 in Slalom task.

Atalante Evolution’s system includes CybAGUI (Cybathlon Atalante Graphical User Interface) application which is provided with a smartphone wrapped around the pilot’s wrist (Figure 7). CybAGUI is an Android application that does not need any network to work, except Bluetooth to communicate with Atalante Evolution. It replaces the standard remote control of Atalante, and provides access to modes which are not available on the regular product (slalom, stairs, … ).

**FIGURE 8:**
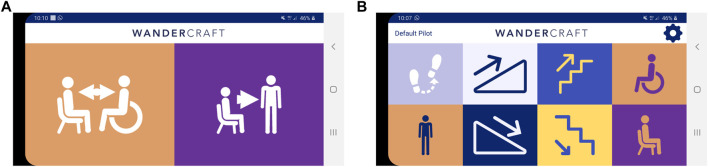
Atalante Evolution’s idle modes (CybAGUI views): **(A)** Sitting mode; **(B)** Standing mode.

The displayed menu changes depending on the current state of the exoskeleton, showing valid transitions. Atalante Evolution has a sitting and a standing idle mode. These modes are crossroads of the state machine to switch from mode to mode. From SITTING mode (Figure 8A), the accessible modes are:

• TRANSFER mode allows the pilot to transfer from his wheelchair to Atalante Evolution, or vice versa, by opening the legs widely.
• STANDUP mode allows the pilot to switch from sitting to standing posture.

From STANDING mode (Figure 8B), the accessible modes are:

• MOVE mode allows the pilot to make steps in six directions. Step length and step number are adjustable.
• RAMP mode allows the pilot to walk up or to walk down Cybathlon ramps with Atalante Evolution.
• STAIRS mode allows the pilot to walk up or to walk down Cybathlon stairs with Atalante Evolution.
• EXERCISE mode allows the pilot to move his upper body to the sides and down while remaining stable and comfortable.
• SITDOWN mode allows the pilot to switch from standing to sitting posture.

For every action, the pilot selects the associated mode on CybAGUI and then, leans forward or on one side to trigger the motion by using the IMU on the jacket that measures the orientation and the velocity of the pilot’s torso.

## 3 Direct-Collocation-Based Optimization Framework

Trajectory generation for humanoid robots is quite challenging. The generation algorithm must handle domains with different contact configurations, provide a trajectory satisfying balance conditions, and respect hardware constraints like joint amplitudes, maximum joint velocities and maximum motor torques. In particular on Atalante Evolution, the joint amplitudes are mechanically limited, not to harm the pilot, and can be more constraining than on other bipedal robotics platforms.

We have the following requirements for our generation framework. It has to provide an efficient way of handling problems with general costs and constraints. The computation time has to be reasonably low, in order to iterate quickly with experiments on the hardware. The framework must be flexible in order to easily tackle different motion tasks and obstacle setups. The framework also has to work with the full model of the robot, so that kinematic and dynamic constraints are taken into account precisely. The direct collocation method (Hargraves and Paris (1987); Denk and Schmidt (2001); Hereid et al. (2016); Harib et al. (2018)) has proven to fulfill these requirements.

Other methods for trajectory generation exist. A common approach for humanoid robots relies on the Linear-Inverted-Pendulum model (LIPM) (Kajita et al. (2003)). In this model, the robot dynamics is approximated by the one of its center of mass (CoM), evolving at a constant altitude. The resulting control system being linear, the trajectory generation problem for the CoM is efficiently solved by a linear-quadratic regulator (LQR). In a second step, trajectories are manually generated for the foot and the pelvis poses, then the full joint trajectory is computed through inverse kinematics. Due to limited joint amplitudes and velocities, this two-step process leads to failures in the generation and restricted motion amplitude. To ensure the comfort of the user of the exoskeleton, we need to add important constraints to the allowed accelerations. This increases the probability of inverse kinematics failures. Moreover, since the full dynamics of the system are not considered (in particular the inertia of the pelvis and legs) in this generation, the final trajectory is not guaranteed to be stable. In practice for Atalante Evolution a very small deviation of the CoM trajectory results in important deviations of the center of pressure (CoP) and thus to theoretically unstable trajectories. Finally, since the generation method is considering only the CoM dynamics and the joint trajectory is computed with inverse kinematics, there is no direct mean to control the motor torques and experimentally, this can result in infeasible motions. Approaches using centroidal dynamics and contact optimization have been proposed, in particular for quadrupeds (Winkler et al. (2018)). They lead to successful trajectory generation on complex terrains, but they don’t allow to put constraints on joint torques nor precise leg kinematics.

Other methods are able to use the full model, in order to take the dynamic effect of all bodies into account. In the single-shooting method, the control input is discretized and the trajectory is found by direct integration of the dynamics equations (Westervelt et al. (2007)). However, this approach is known to be sensitive to initial conditions. Differential dynamic programming iteratively builds and solves an LQR problem, and can lead to efficient trajectory generation (Mayne (1966); Todorov and Li (2005)). However, the method can be sensitive to local minima, and the handling of general constraints is more involved. Multiple shooting can also be a relevant option (Diehl et al. (2006); Schultz and Mombaur (2010); Kudruss et al. (2015)), with performance expected to be close to the one of direct collocation.

### 3.1 System Modeling

The exoskeleton is represented by a kinematic tree with 13 bodies linked by 12 revolute joints, represented in Figure 3. Kinematic and inertial parameters for each link are deduced from the CAD. A kinematic tree with the same structure is generated to represent the pilot’s kinematics. A database of mass distribution for humans taken from (Winter (2009)) provides a relationship between the inertial parameters of each limb. Starting from this data, the pilot’s mass, height and leg segment sizes are used to estimate the mass and inertia of each link. The links representing the pilot are then fused with the ones of the exoskeleton, to provide a dynamic model of the {exoskeleton + pilot} system. We use a floating-base representation of the exoskeleton, with the pelvis link as the root joint. A configuration of the exoskeleton is represented by a vector $q=\left(r_{FF},\phi_{FF},q_{j}\right)\in R^{18}$ , where $r_{FF}\in R^{3}$ is the position of the floating-base, $\phi_{FF}\in R^{3}$ are the Euler angles describing its orientation, and $q_{j}\in R^{12}$ are the angular position of the actuated joints.

An exoskeleton motion is modeled as a sequence of domains with different contact constraints. Examples of contact constraints are: single support on a foot flat on the floor, double support with both feet flat on the floor, rolling contacts around the heel or the toes. In the Cybathlon tasks, the obstacle dimensions are precisely known in advance, so the sequence of foot positions is determined before the definition of the optimization problem. The contact constraints are assumed to be holonomic constraints, which means that they can be characterized as the vanishing of a kinematic function, **f**(**q**) = **0**. Contact forces are represented by a wrench **F** at the contact location. Inside a domain, the system follows continuous dynamics given by.

$$\begin{array}{ccc} D\left(q\right)\ddot{q}+C\left(q,\dot{q}\right)\dot{q}+G\left(q\right) & = & Bu+J{\left(q\right)}^{T}F \end{array}$$ (2)

$$\begin{array}{ccc} J\left(q\right)\ddot{q}+\dot{J}\left(q\right)\dot{q} & = & 0, \end{array}$$ (3)

where $\dot{q}$ and $\ddot{q}$ are velocity and acceleration vectors, *D*(**q**) is the inertia matrix, **G**(**q**) is the gravity contribution, $C\left(q,\dot{q}\right)\dot{q}$ account for inertial effects, *B* is the actuation matrix mapping torques to actuated degrees of freedom, and *J*(**q**) = *∂* **f**/*∂* **q** is the Jacobian of the constraint function. The second equation enforces the holonomic constraint **f**(**q**) = **0** at the acceleration level.

We use a rigid contact model with the floor (Hurmuzlu and Marghitu (1994); Hereid et al. (2016)). When a foot hits the floor, the contact change is assumed to be instantaneous and an impact modeled with a Dirac contact force occurs. During a change of constraints form **f**_*A*_(**q**) = 0 to **f**_*B*_(**q**) = 0, the system undergoes discrete dynamics, given by.

$$\begin{array}{ccc} D\left(q_{I}\right)\left({\dot{q}}_{I}^{+}-{\dot{q}}_{I}^{-}\right) & = & J_{B}^{T}\left(q_{I}\right)F_{I} \end{array}$$ (4)

$$\begin{array}{ccc} J_{B}\left(q_{I}\right){\dot{q}}_{I}^{+} & = & 0, \end{array}$$ (5)

where $q_{I}^{-}$ is the pre-impact configuration, $q_{I}^{+}$ is the post-impact configuration, **F**_*I*_ is amplitude of the impulse force, from which the post-impact velocity ${\dot{q}}_{I}^{+}$ can be deduced.

A particular move is modeled as a motion graph G=(D,E) . A vertex d∈D corresponds to a domain with a fixed set of contact constraints, and continuous dynamics given by Eqs. 2, 3. An edge e∈E represents a transition between two domains *d*_1_ and *d*_2_, including impact dynamics Eqs. 4, 5 if relevant. The transition is modeled by a linkage constraint

$$f_{d_{1}\to d_{2}}\left(q_{d_{1}}\left(T_{d_{1}}\right),{\dot{q}}_{d_{1}}\left(T_{d_{1}}\right),q_{d_{2}}\left(0\right),{\dot{q}}_{d_{2}}\left(0\right),F_{d_{1}\to d_{2},I}\right)=0,$$ (6)

where **q**_*d*_ and ${\dot{q}}_{d}$ are the model trajectories in domain *d*, and $F_{d_{1}\to d_{2},I}$ is the impulse force produced by the impact. In order to be able to execute walk steps in a repetitive manner, the motion graph usually contains cycles. In order to enforce cyclicity constraints, the linkage constraint is modified. For example, to enforce a forward-walking motion, the robot configuration $q_{d_{2}}\left(0\right)$ is replaced by $T_{u}q_{d_{2}}\left(0\right)$ , where $T_{u}$ is the operator translating a robot configuration by a vector **u**. This approach also allows to handle various motions such as displacements in different directions, turning behaviors, standing up and sitting down motions, and to simplify the motion graph by removing redundant symmetric domains. Specific graphs of motions developed during EXO race of Cybathlon 2020 Global Edition are shown in section 4.2.

### 3.2 Optimal Control Problem

Once the structure of the graph has been defined as above, one has to determine the precise shape of the trajectory in each domain. The idea of formulating a motion as the result of an optimization problem has led to successful results (Pandy and Anderson (2000); Hereid et al. (2016)). In general, we consider an *optimal control problem* (OCP) similar to the following:

$$\underset{\left\{T_{d},q_{d}\left(t\right),u_{d}\left(t\right),F_{d}\left(t\right),F_{e,I}|d\in D,e\in E\right\}}{\min}\underset{d\in D}{\sum }\int_{0}^{T_{d}}\left[\alpha_{1}\|{\ddot{q}}_{d}\left(t\right){\|}^{2}+\alpha_{2}\|{\overset{...}{q}}_{d}\left(t\right){\|}^{2}+\alpha_{3}\|u_{d}\left(t\right){\|}^{2}\right]dt,$$ (7)

under the constraints.

• continuous dynamics: Eqs. 2, 3
• impact dynamics/linkage constraints: Eqs. 4, 5 6
• domain duration: *T*_*d*,*L*_ ≤ *T*_*d*_ ≤ *T*_*d*,*U*_
• initial posture: **q**_*d*_ (0) = **q**_0_ for *d* ∈ *D*_0_
• final posture: **q**_*d*_ (*T*_*d*_) = **q**_*f*_ for *d* ∈ *D*_*f*_
• joint limits: **q**_*L*_ ≤ **q**_*d*_(*t*) ≤ **q**_*U*_
• joint velocity limits: ${\dot{q}}_{L}\leq {\dot{q}}_{d}\left(t\right)\leq {\dot{q}}_{U}$

• joint acceleration limits: ${\ddot{q}}_{L}\leq {\ddot{q}}_{d}\left(t\right)\leq {\ddot{q}}_{U}$

• joint torque limits **u**_*L*_ ≤ **u**_*d*_(*t*) ≤ **u**_*U*_
• power limit: $P_{L}\leq {\dot{q}}_{d}^{T}\left(t\right)Bu_{d}\left(t\right)\leq P_{U}$

• friction cone and CoP stability condition: $C_{d}\left(q_{d}\left(t\right),F_{d}\left(t\right)\right)\geq 0$

The precise definitions of the costs and constraints are specific to each task.

### 3.3 Direct Collocation Method

In order to solve the OCP given in Eq. 7, we use the direct collocation approach, in an implementation similar to (Hereid et al. (2016)). The problem in continuous time is *transcribed* into a finite-dimensional non-linear optimization problem. For each domain, we introduce a sequence of *N*_*d*_ + 1 nodes, $0=t_{0}<t_{1}<\ldots <t_{N_{d}}=T_{d}$ . Decision variables are introduced to represent the value of the continuous variables in Eq. 7 at each node. This yields a set of optimization variables $\left\{T_{d},q_{d,i},{\dot{q}}_{d,i},{\ddot{q}}_{d,i},{\overset{\ldots }{q}}_{d,i},u_{d,i},F_{e,I}\right\}$ . To express the relationship between a quantity *x* and its derivative $\dot{x}$ , the integral

$$x\left(t\right)-x\left(0\right)=\int_{0}^{t}\dot{x}\left(\tau \right)d\tau$$ (8)

must be transcribed. This is done by applying a quadrature formula on each interval [*t*_*i*_, *t*_*i*+1_]. We use the Hermite-Simpson scheme.

$$\begin{array}{ccc} x_{i+1}-x_{i} & = & \frac{\Delta t_{i}}{6}\left({\dot{x}}_{i}+4{\dot{x}}_{i+1/2}+{\dot{x}}_{i+1}\right) \end{array}$$ (9)

$$\begin{array}{ccc} x_{i+1/2}-\frac{1}{2}\left(x_{i}+x_{i+1}\right) & = & \frac{\Delta t_{i}}{8}\left({\dot{x}}_{i}-{\dot{x}}_{i+1}\right), \end{array}$$ (10)

where Δ*t*_*i*_ = *t*_*i*+1_ − *t*_*i*_. The Hermite-Simpson scheme requires the introduction of additional times *t*_*i*+1/2_ and the corresponding decision variables *q*_*i*+1/2_, etc. The same quadrature formula is used to evaluate the integral cost in Eq. 7.

All decision variables are stacked into a vector $z\in R^{n}$ . The transcription of the constraints and the cost in Eq. 7 yields the following non-linear optimization problem

$$\underset{z\in R^{n}}{\min}f\left(z\right)\;s.t.\;\begin{array}{c} z_{L}\leq z\leq z_{U}\\ c\left(z\right)=0\\ g_{L}\leq g\left(z\right)\leq g_{U} \end{array}$$ (11)

where $f:R^{n}\to R$ is the objective function, $c:R^{n}\to R^{p}$ is the equality constraint function, $g:R^{n}\to R^{m}$ is the inequality constraint function. In a typical move generation, the number of variables *n*, and the number of constraints *p* and *m*, are of the order of ten thousand. The non-linear program Eq. 11 is solved using the state-of-the art solver IpOpt (Wachter et al. (2005-2021); Wachter and Biegler (2006)). The non-linear problem is solved in less than a minute. Statistics for the optimization problems on the different moves are shown in Supplementary Table S1.

### 3.4 Implementation Architecture

**FIGURE 9:**
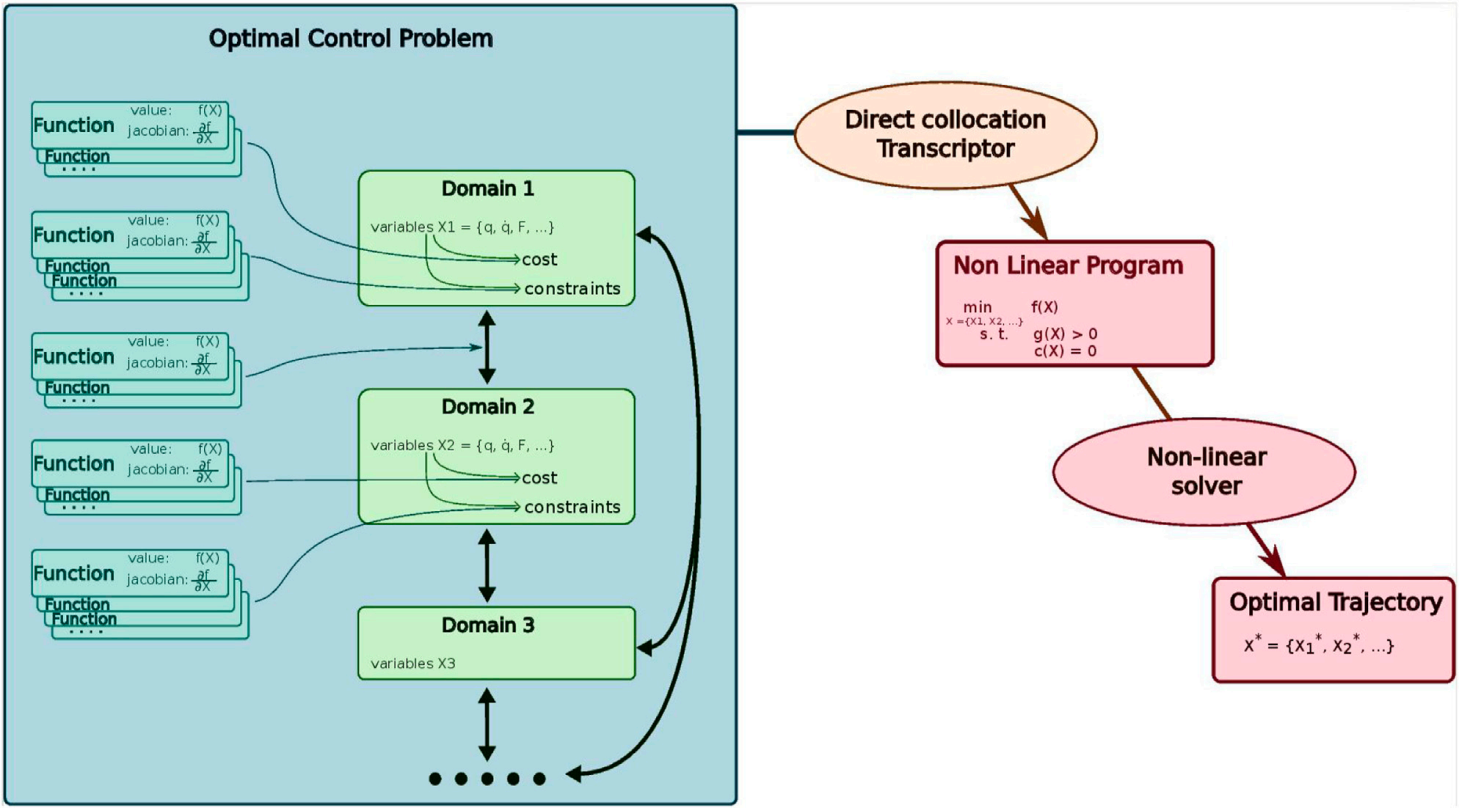
Architecture of our direct collocation implementation.

To ensure fast computation and compatibility with our internal tools, we have implemented our direct collocation library in C++. However, since the trajectory generation is mostly done offline and not directly on the robot, we created bindings in Python. Upon this Python API, we created a wrapper class that allows us not only to quickly define domains and our scenario but also to tune easily the different parameters. There exist two types of parameters: solver parameters which remain unchanged and physical parameters of the motion that we need to tune. The general architecture is summarized in Figure 9. We describe each of our optimization problem in a Python script using this wrapper.

To define an Optimal Control Problem (OCP), we start by defining the list of domains with a fixed set of contact constraints. In each domain we define which variables should be used and the derivative relationships between them. They can be either variables varying with time such as the joint positions or global variables such as the duration of the domain. For Atalante Evolution we usually use **q**, $\dot{q}$ , $\ddot{q}$ , **u**, **F**_*contacts*_ and the duration of the domain.

Once the variables and main contact models are defined in each domain, we need to add constraints and costs to actually define which motion we want to execute. The main goal of our architecture in this step is to make it easy to write and read, with as much flexibility as possible. This was achieved with our main component: the *Function* class. A *Function* defines a holonomic constraint or an objective. As the name suggests, for a given input variable, it computes a value. This value can be used as cost and minimized or as a holonomic constraint and thus be required to be equal to a certain value or be in a given interval. In addition, it also computes the associated Jacobian matrix which is necessary for fast optimisation.

A *Function* can be simple, for instance, it can enforce a variable to a certain value. But it can be more complex. For instance, we defined several system-specific *Functions* to enforce the position of the CoP or of the feet of the robot. Most of the *Functions* specific to Atalante Evolution are highly non-linear and even non-convex. They are implemented with the fast C++ Dynamics library Pinocchio (Carpentier et al. (2015-2021); Carpentier et al. (2019); Carpentier and Mansard (2018)). The necessary Jacobians are computed analytically. In particular, Pinocchio provides partial derivatives of kinematic and dynamic functions.

For each domain we can then use our *Function* library to apply constraints or add costs to each domain. To do so, we can choose to apply them on the variables all over the domain: e.g., “Pelvis vertical velocity should never go above 3 cm/s during all the domain.” We can also apply the function only at some points of the trajectory (defined by percentage of the domain duration): e.g., “Left foot should be above 4 cm at 50% of the domain.” This strategy is in particular useful to define the constraints between two domains. We can indeed define some continuity constraints between the variables at the end of a domain and the variables at the beginning of another one. But more complex transitions are possible, for instance for the transition between a left and a right step in the walk pattern, we define a *Function* to enforce the following constraints:

• The floating-base has moved forward by the desired step length
• The joint positions, velocities and accelerations are symmetrized.

Once the OCP is correctly defined, a direct collocation transcriptor will transcribe it into a Non-Linear Program that can be solved with IpOpt [Wachter et al. (2005-2021), Wachter and Biegler (2006)] using MA27 linear solver. To do so, the variables varying with time along a domain are discretized according to section 3.3. The cost and constraints are applied either to a specific node or to all the nodes of the domains if required.

## 4 Motions Generation and Pilot-Exoskeleton Cooperative Strategy

### 4.1 Introduction of the Trajectory Generation Process

In the Cybathlon tasks, the first step has been to identify the different motions that were necessary. Many of them were not implemented on the commercial version of Atalante. Among them, some are quite obvious such as climbing steps or ramps, or standing up from a low sofa. However, other fine displacements, such as side steps, were also necessary to position the exoskeleton precisely along the obstacles and accomplish all the tasks.

For each movement, we defined the sequence of domains to execute. Each domain has a constant set of contact constraints (see section 3). The most common types are *double support* phases where both feet are flat on the floor and *single support* where one foot is flat on the floor and the other foot is moving. A simple displacement is a succession of these types of domains. But there can be a more complex contact set. For instance for going down stairs, in some domains one foot is flat on the step and the other rolls around a given edge.

We have defined common basic sets of constraints for each type of contact model. In particular, the physical constraints of Atalante Evolution exoskeleton such as torque and joint limits are included. This allows us to create the basis of any scenario quite efficiently. Then we can add constraints in each domain and between them to enforce the actual motion that we want.

When our problem is correctly defined, we can write down the domains with their respective constraints and cost in a Python script using the interface we have described in the previous sections. When the solver has converged, we have several graphical tools to analyse the result before actually testing it on the exoskeleton. In particular, we use the graphical robot viewer developed by the *Gepetto* team of the *LAAS* (Mirabel et al. (2014–2021)) to watch the trajectory.

In each movement that we generate, we choose some physical parameters arbitrarily, for instance the maximum foot height during a step, or the duration of a double support phase. We need to find the parameters that guarantee the full autonomy of the pilot while efficiently satisfying the task requirements. Depending on the complexity of the task, the number of parameters can go from 2 to 10. In order to find those parameters, we have an experimental phase during which we will iteratively test different sets of parameters. In each iteration, we update the parameters and we check the result with our graphical tools. If satisfied, we test it on the exoskeleton with an able-bodied subject, and then we iterate with our pilot when the trajectory is mature enough. The goal is to find stable, robust and comfortable movements for the pilot.

The development time for a new gait varies depending on its complexity. However, we can estimate to around 1 week the typical time necessary to write down a complex optimization problem and another week to test it on the exoskeleton and adjust the constraints and parameters to the reality with the paraplegic pilot.

For the different motions, we have computed key performance indicators (KPIs) to show how they perform. Those indicators are taken from the Memmo project’s benchmarking criteria (Morchionni (2018)) based on Torricelli et al. (2015). We have computed on relevant motion sequences the following indicators:

• Duration of the sequence *T*_*M*_ = *t*_*end*_ − *t*_*begin*_,
• Travelled distance *D*,
• Velocity *v* = *D*/*T*_*M*_,
• Forces measured by the feet sensors,
• Cost of transport $CoT=\int_{t=0}^{T_{M}}\left(E_{mechanical}+E_{electrical}\right)\;dt/G\;M\;D$ which is a dimensionless ratio and defines the energy efficiency to travel the sequence, with *M* the mass of the system exoskeleton with its pilot,
• Froude number $F_{r}=v/\sqrt{gl}$ (also called dimensionless gait velocity) which represents the ratio between the kinetic energy and the potential energy and is used to study dynamic walking patterns similarities between robots and animals of different sizes, with *g* the gravitational constant, and *l* the leg length.

Those values are only considered as indicators. They are not given as cost in the optimization but indirectly optimized. We indeed try to find the fastest possible gait with the hardware, stability and comfort limitations detailed in the next subsections. Likewise, the energy is indirectly minimized by the cost used in the optimization on the motor torques.

### 4.2 Motions Generation for Cybathlon’s Tasks

#### 4.2.1 Sit and Stand Motions

The first obstacle of the race consists of sitting on a sofa then standing up (see Figure 2A). When seated, the CoM of the robot with user is behind the heels. When standing up, the polygon formed by the feet becomes the stability polygon. Statically, with no external force, it is therefore not possible to stand up. Moreover, the sofa is much lower than the standard seat we use for our product and the pilot can only help the motion with one armrest. We worked on this generation problem to find trajectories that keep the robot balanced while standing up using the dynamics and the help of the pilot.

To ensure the motion is balanced, we choose to use the CoP as cost of the optimisation all along the motion. With this cost, the dynamic effects will move the CoP forward and bring it as fast as possible inside the stability polygon of the feet. On the other hand, the external force provided by the user is not added to the model. Moving forward the CoP toward the support polygon will indeed result in a reduction of the user’s efforts.

**FIGURE 10:**
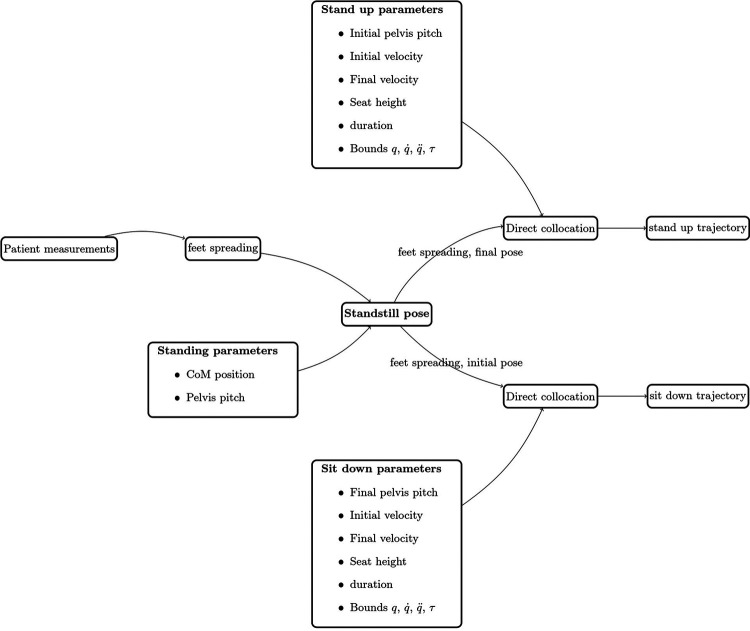
Stand and sit trajectory generation framework.

The trajectory generation starts with generating a standing posture, which will be the initial (resp. final) posture of the sit-down (resp. stand-up) trajectory and define the feet position - which shall not move during the trajectory. This posture depends on the patient leg measurements. The taller the patient, the more the feet will be spread. Some additional parameters to define the initial and final conditions or the joint bounds are added as constraints to the direct collocation problem. Figure 10 defines the state machine schema of the sit-down and stand-up trajectory generation.

During the training sessions, we have iterated on several parameters to improve the quality of the motion and decrease the user effort necessary to keep the exoskeleton balanced. In particular, we had to find a compromise between the different costs: CoP, torque and acceleration. The initial pelvis pitch velocity is also an important parameter as it synchronises the robot with the user pelvis impulse when triggering the motion.

**FIGURE 11:**
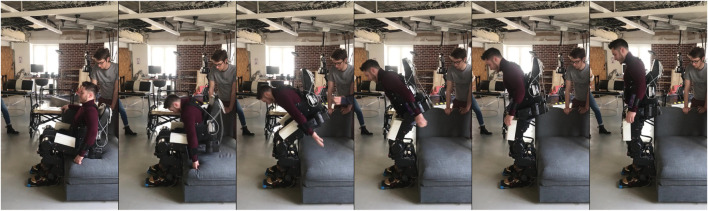
Pilot standing-up from the sofa.

The result of the motions executed by our Cybathlon pilot is visible in Figure 11 and Supplementary Figure S1.

#### 4.2.2 Multi-Directional Steps

During all the tasks, we need to be able to position precisely the exoskeleton. The other challengers rely heavily on their crutches to do so. The pilots use their arms to transfer their weight on one leg and then use their torso to trigger the desired displacement. We can see the teams do with this technique 180°turns, some back or side steps.

Without crutches, we need the exoskeleton to do the same fine displacements on its own. At the time the Cybathlon race took place, the only movements already generated in the commercial version of Atalante were a rotation movement and forward straight walk. We therefore needed to evaluate which movements were necessary to implement for the competition, assess which ones were possible to execute on the robot and finally generate and tune each of them.

We explored the possibility to have steps in 8 directions (forward, backward, sideways and the 4 associated diagonals). However, having so many directions turned out to be difficult to control and learn for the pilot. It brought a lot of mental load and a small gain in time in the different tasks. We finally decided to use forward, backward and side displacements only, in addition to the rotations.

We also realized that we usually had two opposite objectives. On the one hand, we need to go fast to realize each task in the minimum of time. For this objective, we need long steps with a high cadence. On the other hand, when coming close to an obstacle or reaching a spot to start another motion, we must be as precise as possible. For the specific case of the back steps, we also needed to walk backward slowly when passing through the door to catch the handle and close the door.

For the forward displacement, the walk movement is already present and optimized on the robot. So we will focus in this section on the other movements.

Each displacement is be generated as a sequence of domains with the two feet (flat) on the floor and domains with only one foot (flat) on the floor. During the domains with two feet on the floor, the exoskeleton transfers its CoP toward the foot which is the next support foot. During the domains with only one foot on the floor, the robot makes steps in the required direction.

Let us first focus on the back steps. In this trajectory, the robot first executes a starting right step. During this step, its right foot will move backward by half of the desired step length. Then the robot will execute alternatively left and right steps which move the appropriate foot backward by the desired step length. When required by the user, the exoskeleton will execute a stopping step which brings the two feet back to their final positions and stops the displacement.

The generation is done according to the state graph in Supplementary Figure S2. We can emphasize the fact that we exploit the symmetry of the problem and do not explicitly generate the trajectories for all the domains. We generate the domains in green in Supplementary Figure S2. To simulate the domains in white we add a specific constraint between the “cyclic left step” and the “cyclic weight transfer to left” domains. This constraint specifies that the positions and velocities of the joints should be continuous with their symmetric counterpart. This allows to generate fewer domains, but finally have the full movement by symmetrization of the generated domains. Besides, this ensures that we generate a symmetrical walk.

The result of the movement executed by our Cybathlon pilot is visible on Supplementary Figure S3.

The lateral displacement cannot follow this pattern because the displacement is not in the sagittal plane. If we take the example of side steps to the right, the exoskeleton will first realize a right step during which it will move its right foot to the right. Then it will bring back its left foot close to the right one. It will repeat this pattern until the pilot orders to stop.

The displacement to the left is done with the same pattern symmetrized. For the rotations, the step pattern will be the same but during the first step, instead of moving sideways the foot will be required to turn.

The generation is done with the state graph in Supplementary Figure S4. We can note that here we need to generate all the domains as there is no symmetry. But we can generate only one displacement direction and symmetrize it to obtain the displacement in the opposite direction.

The result of the movement executed by our Cybathlon pilot is visible on Supplementary Figure S5.

With those patterns decided, we can generate trajectories and start the parameters tuning phase on Atalante Evolution. The objective is to find the trajectories which require less cooperation efforts (or even no effort at all) from the pilot. The main challenge for tuning the parameters originates from the robot’s structural deformations that occur during the trajectory. Main deformations have been identified to occur at the level of the ankles and the hips (Vigne et al. (2020)). They have two main effects:

• The CoM does not follow the expected trajectory, thus harming the predicted stability of the movements.
• The steps are not fully executed and the impacts are detected before the end of the generated trajectory and the controller switches to the next domain earlier than planned.

To compensate for those effects, we add offsets on the generated centroidal trajectory to increase the stability. We also constrain the foot trajectory at the end of the steps. The goal is to have smooth impacts (both for stability and comfort) and prevent the foot to slip on the floor at the domain transition.

Another objective when tuning the parameters is to have a movement that is comfortable for the pilot. One example of parameters tuned with this objective is the duration of the weight transfer phases between two steps. We initially set those domains to have a duration of around 0.5 s. However, the experiments showed that so long weight transfer phases gave an impression of feet glued to the floor. To have an impression of a real dynamic motion, we needed to reduce the duration to 0.1 s.

Some key performance indicators about the back steps, side steps and rotations can be found respectively in Supplementary Tables S2, S3, S4.

#### 4.2.3 Slalom

In certain situations in daily life, it is required to move around obstacles in order to avoid collisions with obstacles on the path or to reach a certain destination. The slalom task is a challenge from the EXO race during which the pilots have to negotiate a slalom composed of three tables plus a coat rack aligned with 1 m distance between each of them, see Figure 2B. The state machine of the slalom walking gait is shown in Supplementary Figure S6. Only the 7 phases in green are generated, and the other phases are obtained by the matching phase. The walking specifications such as stride length of 30 cm, step width of 28 cm and step duration of 1 s are set on the basis of experimental results with the pilot. A longer stride length is observed to be less stable. A shorter step duration makes the walk less comfortable and more difficult to keep stable for the pilot because of the higher frequency of stronger impacts with the floor. The step width is chosen to avoid the collision of the two leg shells during the walk. The swing foot clearance of at least 6 cm is set at the mid-step. The impact of the swing foot with the floor has been regulated with near-zero forward and lateral velocities to limit the transmission of vibrations from the exoskeleton to the pilot which causes an uncomfortable sensation. For the turning walk, no constraints imposed the radius of curvature in our approach because a predefined radius of curvature is usually unknown in almost every real situation. Therefore, the only constraint on the torso yaw is set to 30°after two domains of the turning walk. This value is chosen to achieve a stable and comfortable turning walk in the limit of the transverse hip joint. The pilot-exoskeleton cooperative strategy is a key to accomplish the slalom task. The pilot can modify the radius of the turning motion by putting his weight on the support leg or on the swing leg. He also decides the turning direction, when and how many times to turn in order to avoid the obstacles via CybAGUI according to the situation. Supplementary Figure S7 illustrates the autonomous navigation of our pilot to accomplish the slalom task. Some key performance indicators can be found in Supplementary Table S5.

#### 4.2.4 Stairs

Stairs are very common in the urban environment. The Stairs task is a part of the challenge from the EXO race for which the pilot must control the exoskeleton to ascend and descend a 6-steps staircase with a step height of 17 cm and a step length of 28 cm, see Figure 2C. Since Atalante Evolution’s foot measures 37 cm long whereas the stair is 28 cm long, only a part of the foot can be on the stair. Moreover, the ankle joint range and torque limits plus the fact that the weight of the robot is at the back, make the stairs difficult to ascend and descend in autonomy. To overcome these difficulties, the pilot-exoskeleton cooperative strategy is integrated into the generation. The pilot has to help to adjust the placement of the foot when landing on the stair and make the upstairs/downstairs walking more stable by using the handrails. For the security of the pilot, we chose to execute the upstairs walking forward and the downstairs walking backward. The pilot can position itself in front of the stairs correctly by making one or several steps of a generated walking gait with small step length of 5 cm. The walking is also stopped when no contact is detected between the foot heel and the stairs when positioning at the top of downstairs. The different walking gaits have been generated with 7 domains as shown in Supplementary Figure S8 for upstairs walk and with 14 domains as shown in Supplementary Figure S9 for downstairs walk. The swing foot is imposed to be move vertically at the beginning and the end of each step in order to avoid hitting the stair. The weight transfer double support domain has a role as a temporal phase that allows the pilot to prepare for the next stair. The stopping phase is composed of several steps in order to go away from the final stair for the security of the pilot. A domain during which the foot rolls on its toes is added into the downstairs walk to make the walking more natural and to reduce the pulling-up effort of the pilot at the end of each step. The effort of the pilot is also taken into account in the optimization parameters tuning as a constraint and is evaluated during the training. Supplementary Figures S10, S11 illustrate the autonomous navigation of the pilot to accomplish the Stairs task. It is noted that the stopping phase for Stairs task is automatically done by the control algorithm when the number of steps is reached, i.e., *n*_*steps*_ > 6. The key performance indicators to monitor the progress and benchmark of the pilot-exoskeleton cooperative strategy for stairs task validation can be found in Supplementary Tables S6, S7. The CoT for upstairs walk (218.407) is bigger than the one for downstairs walk (67.774). This indicates that the pilot needs to provide a bigger effort for walking upstairs, which is consistent with the experimental results we observed.

#### 4.2.5 Ramps

Inclined surfaces such as ramps are a part of a natural path in daily life. In Ramps task, the pilot must ascend on a slope of 20°, open, pass and close a door and descend on a slope of 15°, see Figure 2D. This task is a real challenge for Atalante Evolution exoskeleton due to the limitation of the sagittal ankle joint. Many strategies have been developed and tested with the pilot. Finally, going up the ascending ramp in the backward direction and going down the descending ramp in the forward direction have been chosen to satisfy requirements on the stability and pilot security. The different walking gaits have been generated with 11 domains for ascending ramp, see Supplementary Figure S12 and with 10 domains for descending ramp, see S13. The number of domains of walking gaits is chosen to ensure the smooth transition. The major challenge of this task is to ensure the self-balance of the pilot with the exoskeleton for the change of the ground-feet contact angle at the beginning and at the end of the ramp. The pilot-exoskeleton cooperative strategy has a key role to overcome this difficulty. In order to ensure the success of the ascending or descending walk, the pilot shall position himself right in front of the beginning of the ramps with the feet toe align with it. The pilot can also make the walking more stable and control the direction by using the handrail.

For the walk on ascending ramp in backward direction, the pilot cannot see when he arrives at the top of the ramp. To overcome this problem, a contact detection algorithm has been implemented in which the walk automatically pauses at the top of the ramp when the heel is not in contact with the ramp anymore and a minimal number of steps has been reached, i.e., *n*_*steps*_ > 16. This value is defined from experiments. The pilot can decide his position at the top of the ramps after the automatic stop via CybAGUI by executing more steps (position he learned by training) before triggering the stopping phase.

For the walk on descending ramp in forward direction, the pilot makes the decision of executing the stopping phase when arriving at the end of the ramp. Supplementary Figures S14, S15 illustrate the autonomous navigation of our pilot to accomplish the Ramp task. As observed from experiments, the pilot needs to provide more effort for the ascending ramp walk with respect to the descending ramp walk. This fact can be verified with the CoT (see Supplementary Tables S8, S9) for both trajectories. We can see that indeed the ascending ramp walk has a higher cost (123.7) than the descending ramp walk (48.9).

## 5 Conclusion

Wandercraft completed the development of new features to perform 4 tasks out of 6 at the EXO race of Cybathlon 2020 Global Edition with Atalante Evolution - the only crutch-less powered exoskeleton presented at the race–in under a 12 months timeline. It was a real challenge to adapt our exoskeleton to Cybathlon’s requirements since the robot was intended for rehabilitation centers with a physiotherapist to supervise the patient. For the race, our pilot Kevin Piette equipped with Atalante Evolution, had to perform the tasks in full autonomy, with four spotters around him in case of unbalanced situations or failures of the robot (section 2).

Designing new dynamic motions with an 82-kg exoskeleton on the pilot and taking the least possible risks for his physical security (e.g., walk with reduced speed), was possible thanks to the direct-collocation-based optimization framework we developed these past years (section 3). We improved the tools and the process of trajectory generation to be able to address quickly any new task. A large part of the work consisted of testing with the pilot to have feedback on the feasibility and the success of the motion: evaluating the impacts of the deformations of the mechanical structure of the robot and analyzing the required attitude of the pilot while moving (section 4). All this feedback was integrated into the optimization formulation to improve the motions at the robot side, as much as possible. Finally with training, Kevin was able to adjust himself to stabilize the different motions and to realize the tasks. This framework is also used to generate trajectories for different patients who use Atalante in their rehabilitation program. For the commercialized version of Atalante, we designed several trajectories with this same framework and we test the generation on a set of 1,000 different patients with a failure rate around 0.1%.

As result, we performed the 4 tasks successfully in 7 min 26 s (see videos on Cybathlon’s website) in complete autonomy. We were ranked 8th, but we were highlighted as being the future of lower-limbs exoskeletons. This challenge was a real success for Wandercraft: we learnt a lot both on Atalante’s hardware limitations and on how to improve the features to fit with daily life. The new features we made, have improved our robots in different ways: some of them are ready to be integrated in the product Atalante for patients in rehabilitation centers such as multi-directional steps and sit-and-stand from any height of chair. The rest of the new features are more complex (i.e., non-standardized step height or slope angle in the urban environment) and make more sense for the development of the next generation of Wandercraft’s exoskeleton: the personal and outdoor exoskeleton with a reduced mechanical structure and a better actuation technology to face any obstacle.

## Data Availability

The original contributions presented in the study are included in the article/Supplementary Material, further inquiries can be directed to the corresponding author.
