# Supplementary figures and images for: Versatile Dynamic Motion Generation Framework: Demonstration With a Crutch-Less Exoskeleton on Real-Life Obstacles at the Cybathlon 2020 With a Complete Paraplegic Person
Source: Front Robot AI. 2021 Sep 24;8:723780. doi: 10.3389/frobt.2021.723780 (PMC8498038; doi:10.3389/frobt.2021.723780)

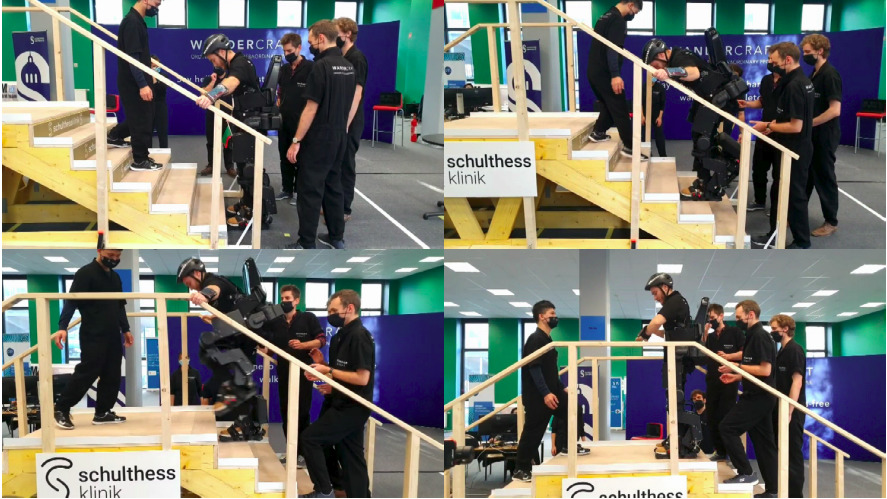

Supplement: Supplementary file 2 [file DataSheet1.zip › Data Sheet 1/Supplementary Images/S10-upstairs_exp.jpeg]

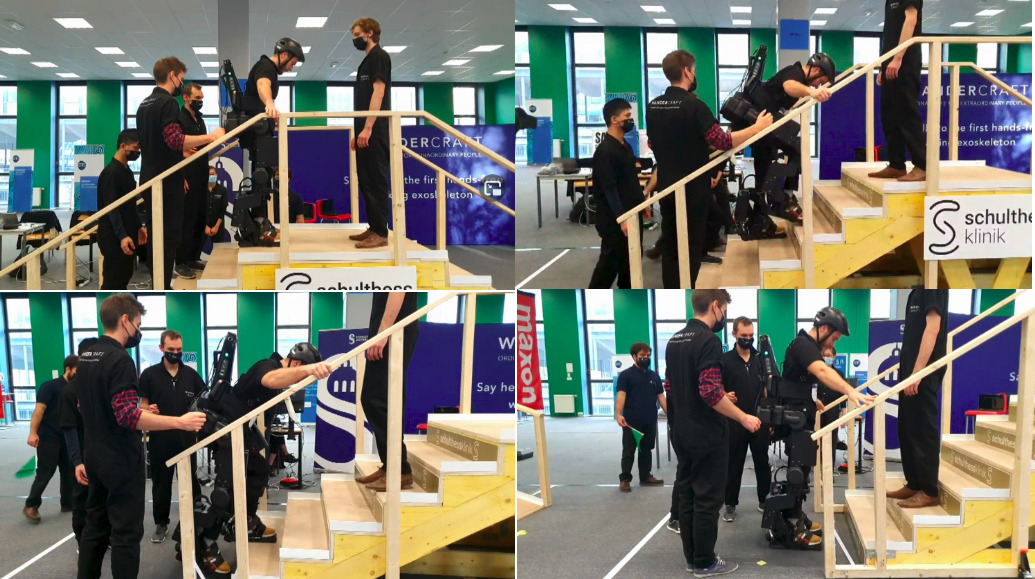

Supplement: Supplementary file 2 [file DataSheet1.zip › Data Sheet 1/Supplementary Images/S11-downstairs_exp.jpeg]

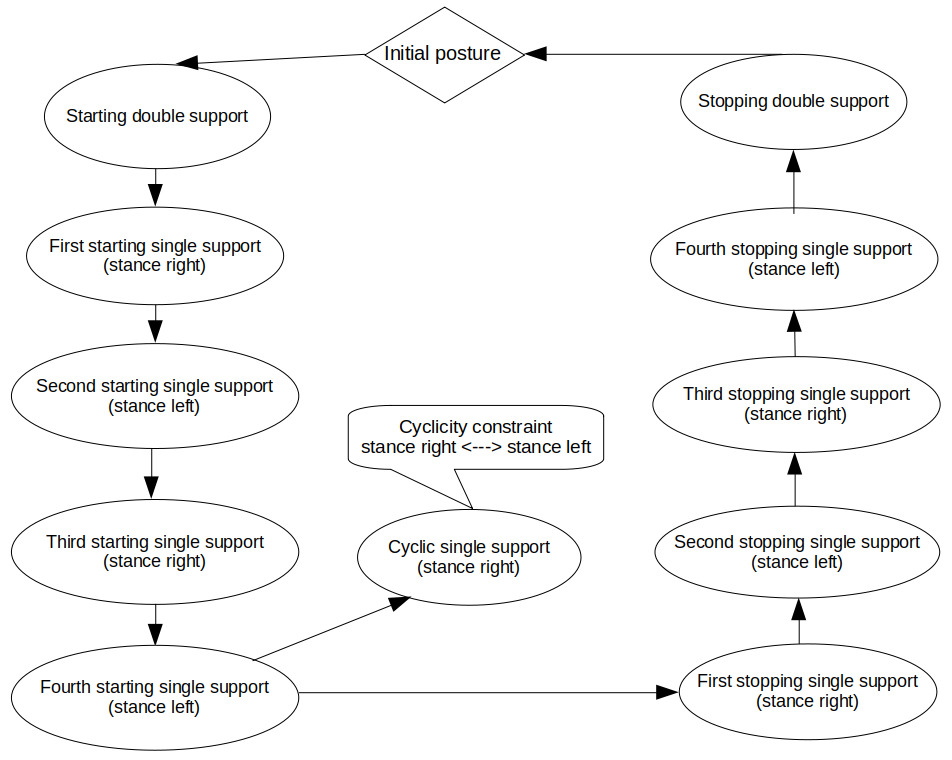

Supplement: Supplementary file 2 [file DataSheet1.zip › Data Sheet 1/Supplementary Images/S12-asc_ramp_schema.jpeg]

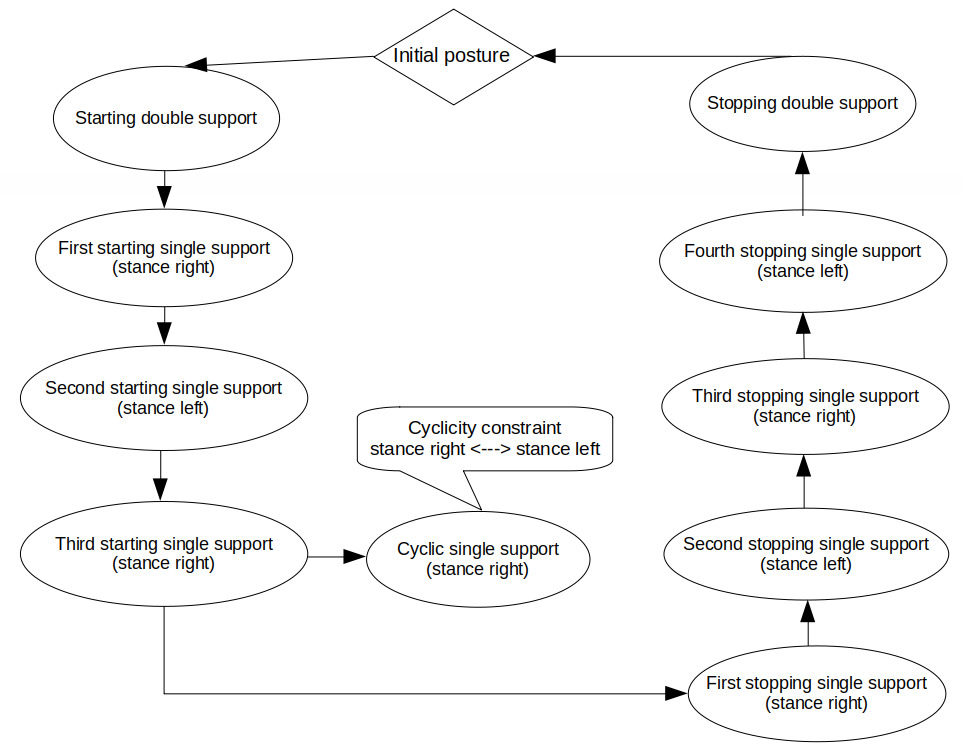

Supplement: Supplementary file 2 [file DataSheet1.zip › Data Sheet 1/Supplementary Images/S13-dsc_ramp_schema.jpeg]

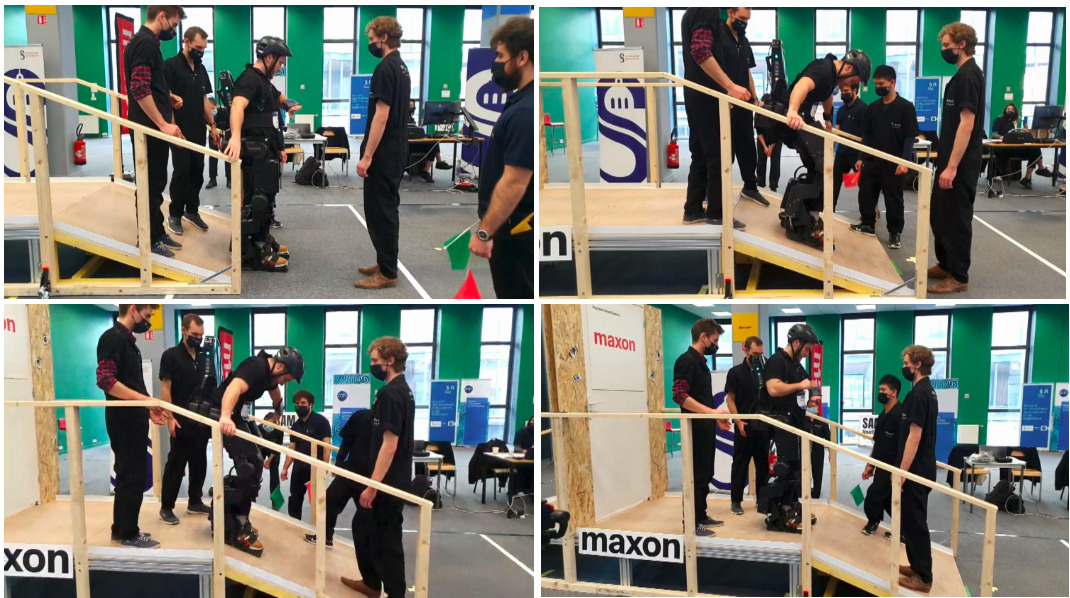

Supplement: Supplementary file 2 [file DataSheet1.zip › Data Sheet 1/Supplementary Images/S14-asc_ramp_exp.jpeg]

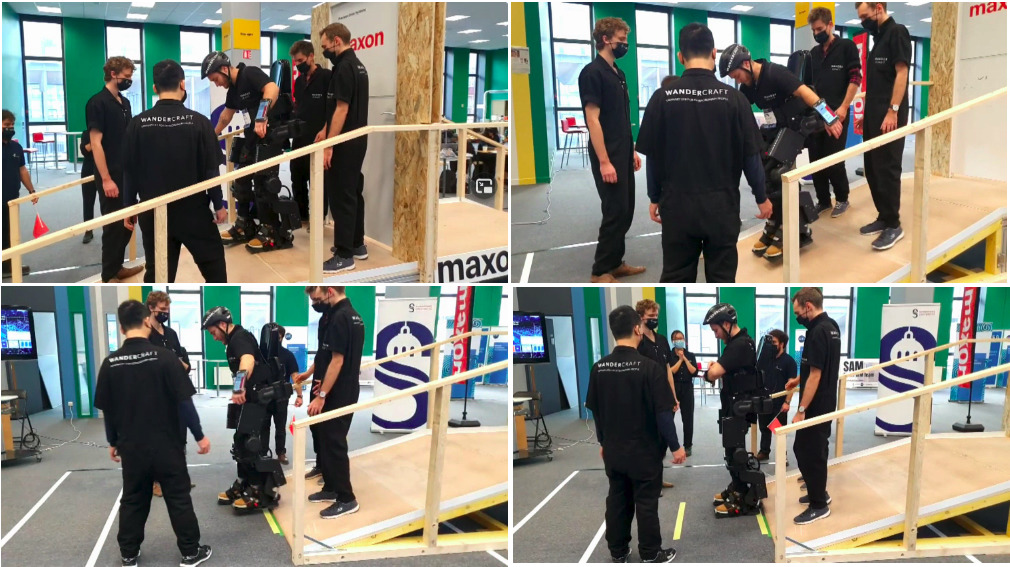

Supplement: Supplementary file 2 [file DataSheet1.zip › Data Sheet 1/Supplementary Images/S15-dsc_ramp_exp.jpeg]

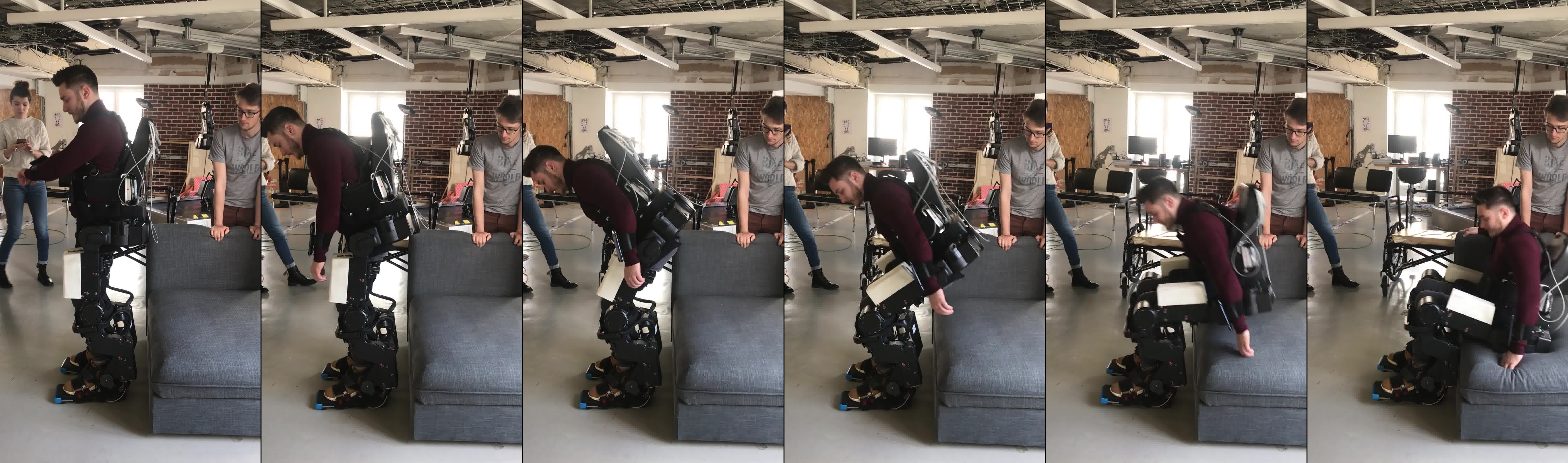

Supplement: Supplementary file 2 [file DataSheet1.zip › Data Sheet 1/Supplementary Images/S1-frames_sit_down.jpeg]

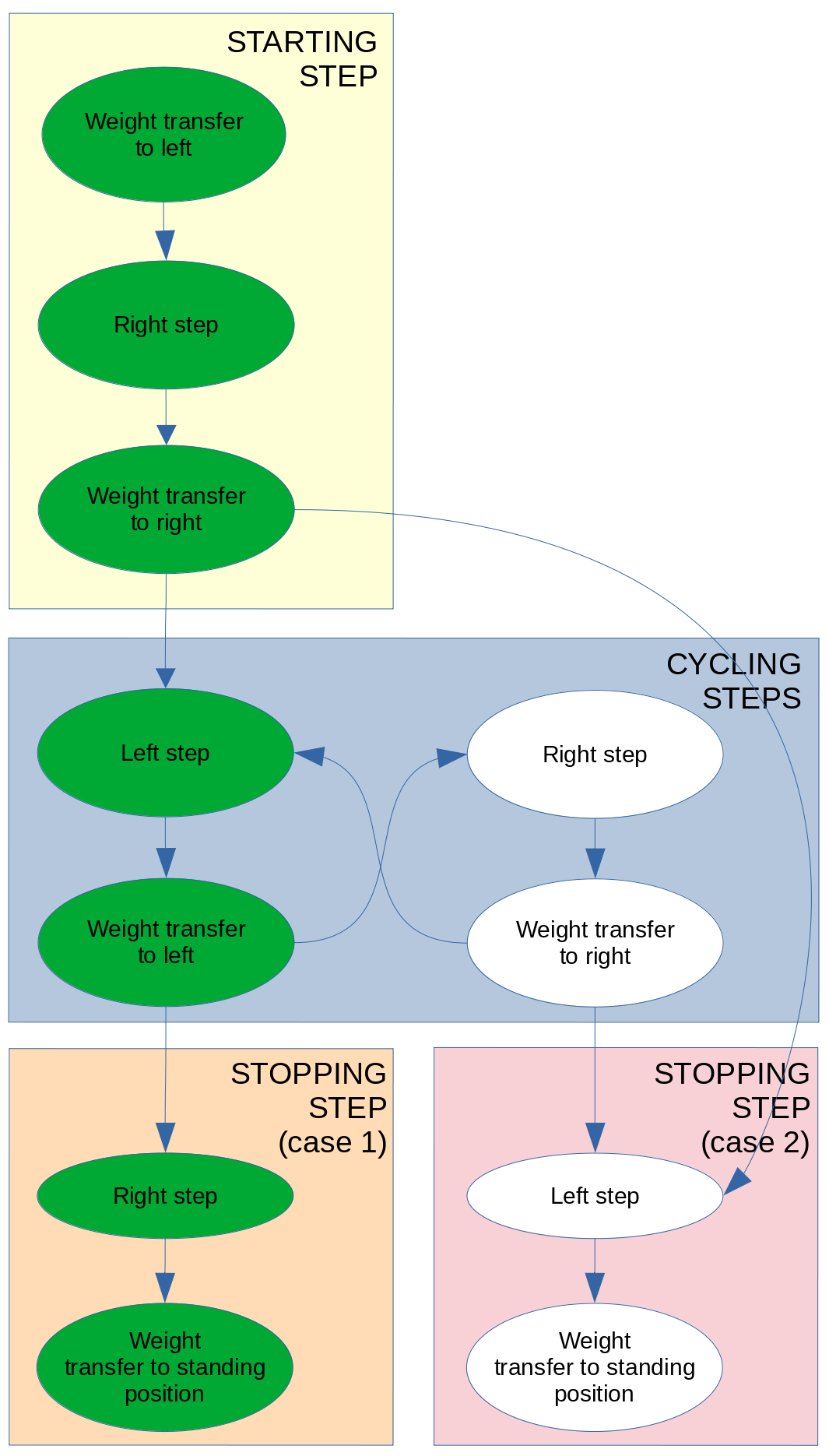

Supplement: Supplementary file 2 [file DataSheet1.zip › Data Sheet 1/Supplementary Images/S2-backsteps_state_machine_cybathlon.jpg]

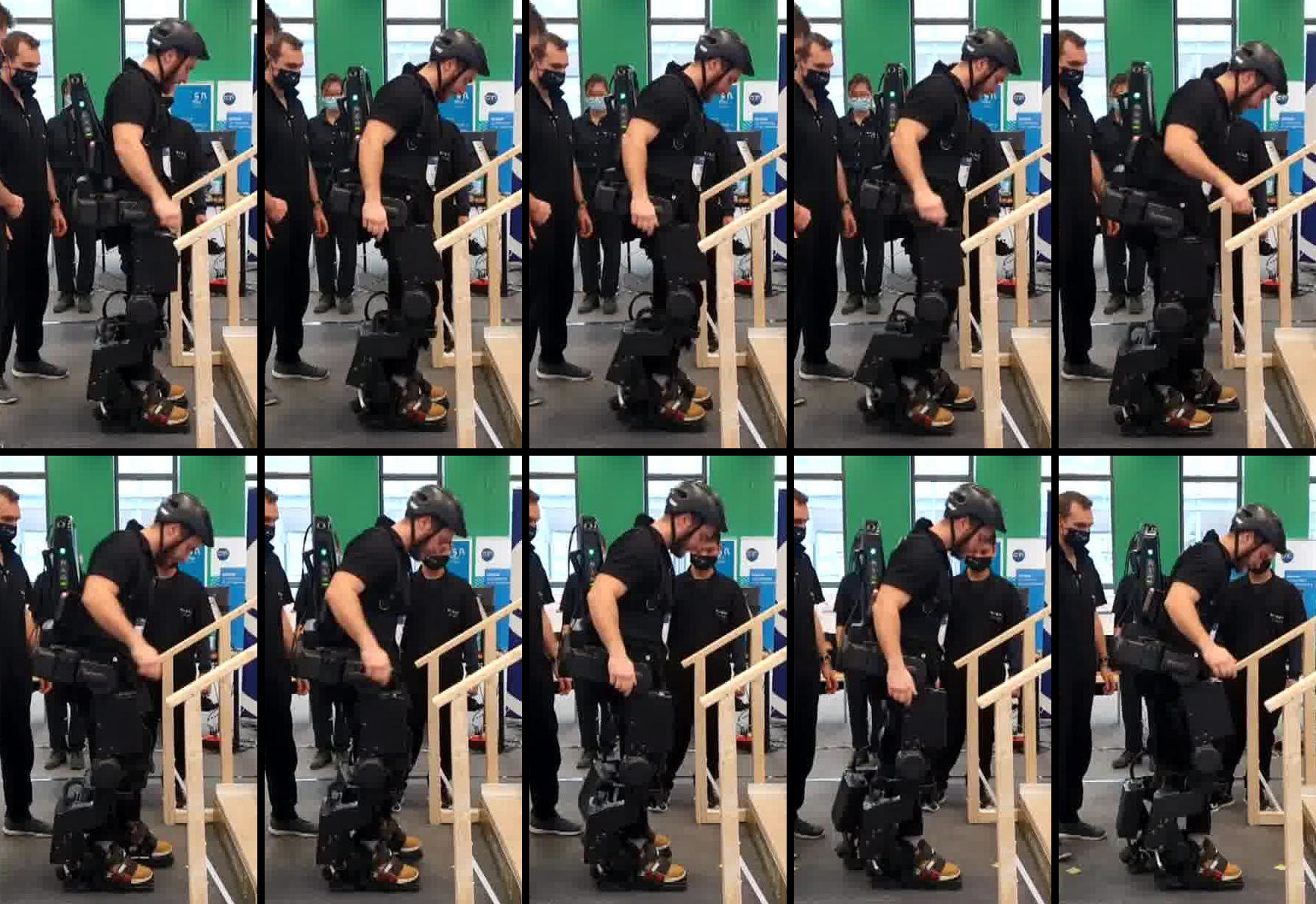

Supplement: Supplementary file 2 [file DataSheet1.zip › Data Sheet 1/Supplementary Images/S3-backsteps_img_tiles.jpeg]

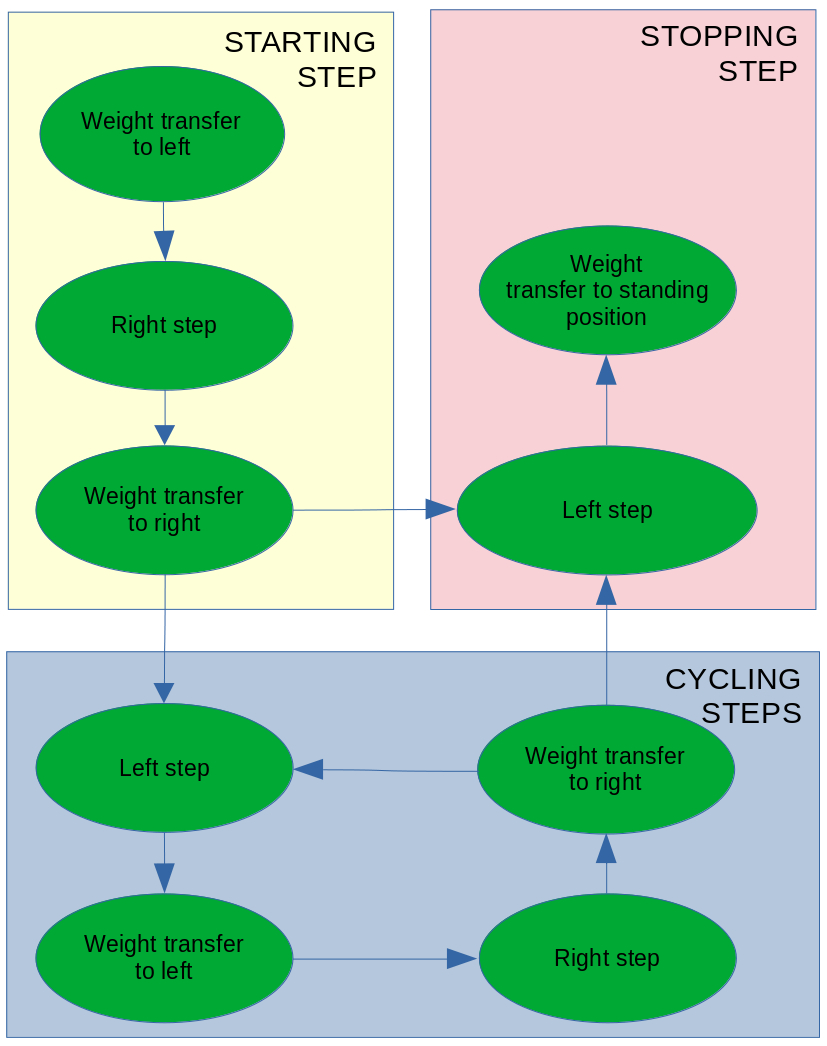

Supplement: Supplementary file 2 [file DataSheet1.zip › Data Sheet 1/Supplementary Images/S4-sidesteps_state_machine_cybathlon.jpg]

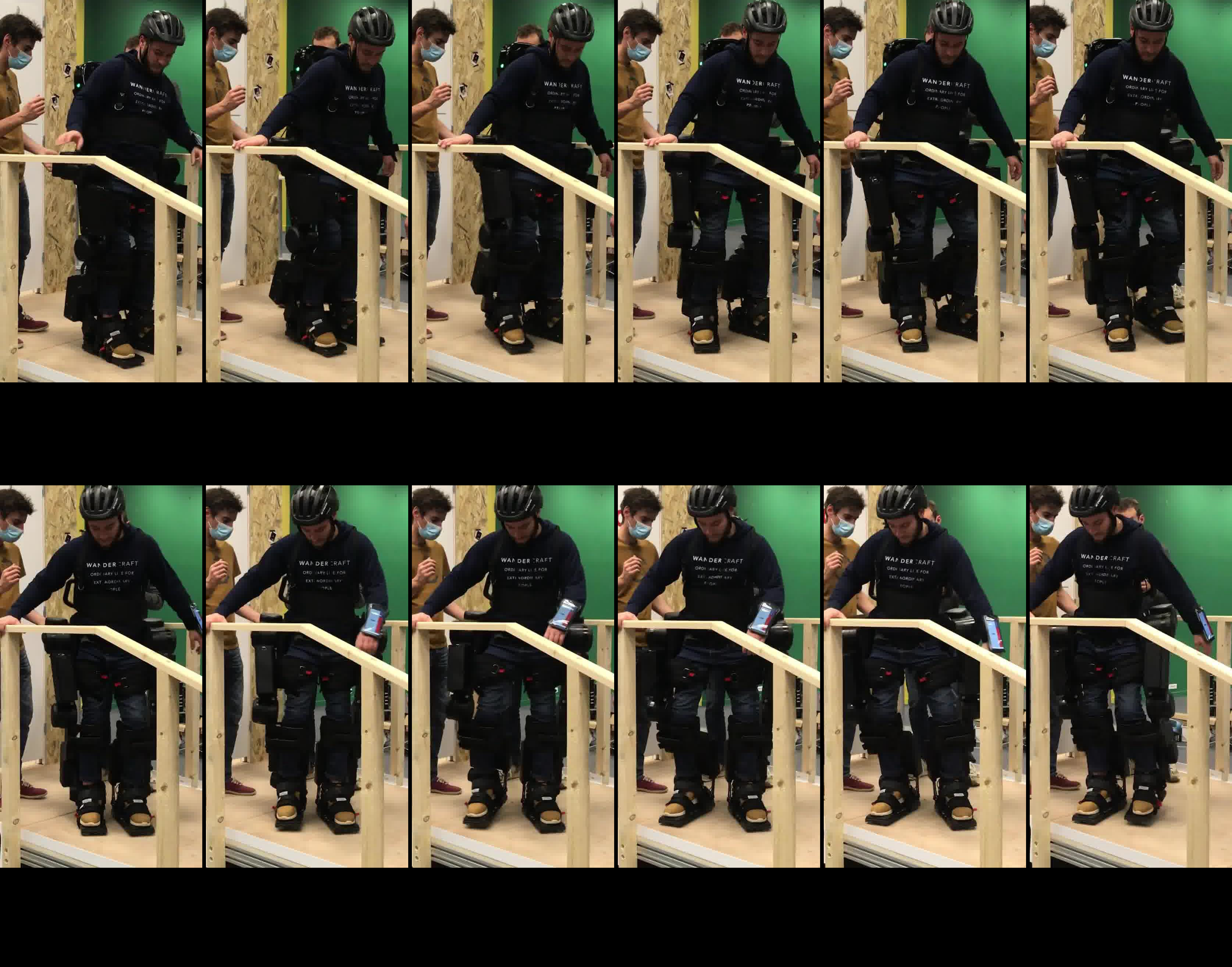

Supplement: Supplementary file 2 [file DataSheet1.zip › Data Sheet 1/Supplementary Images/S5-turn_img_tiles.jpeg]

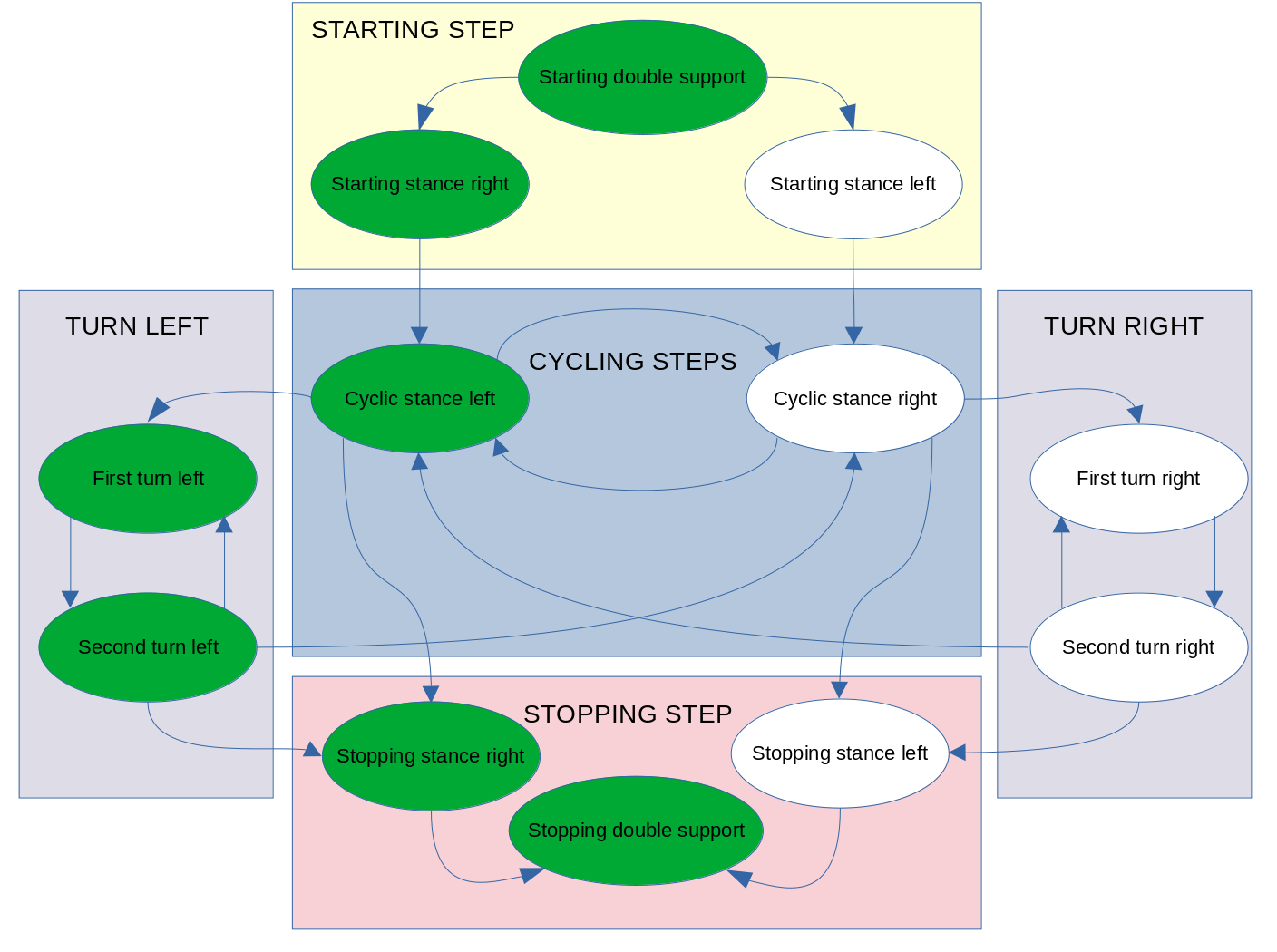

Supplement: Supplementary file 2 [file DataSheet1.zip › Data Sheet 1/Supplementary Images/S6-slalom_state_machine.jpg]

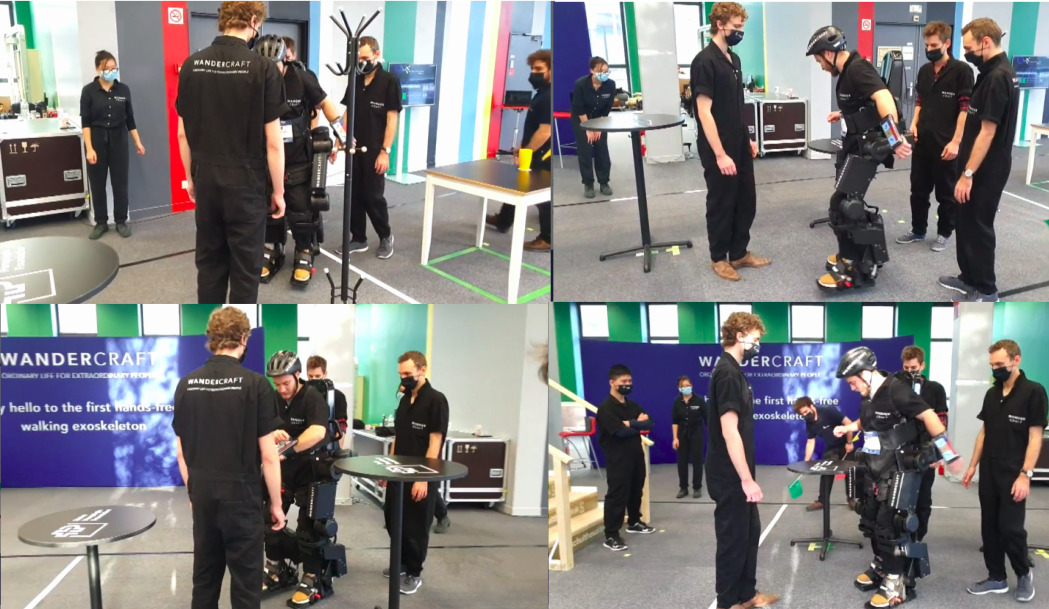

Supplement: Supplementary file 2 [file DataSheet1.zip › Data Sheet 1/Supplementary Images/S7-slalom_exp.jpeg]

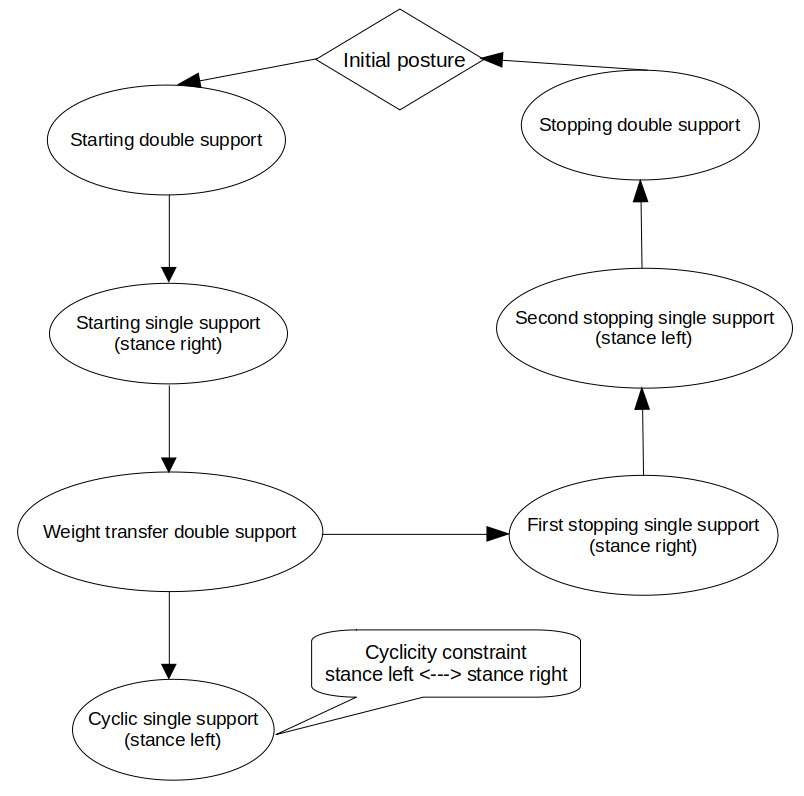

Supplement: Supplementary file 2 [file DataSheet1.zip › Data Sheet 1/Supplementary Images/S8-upstairs_schema.jpeg]

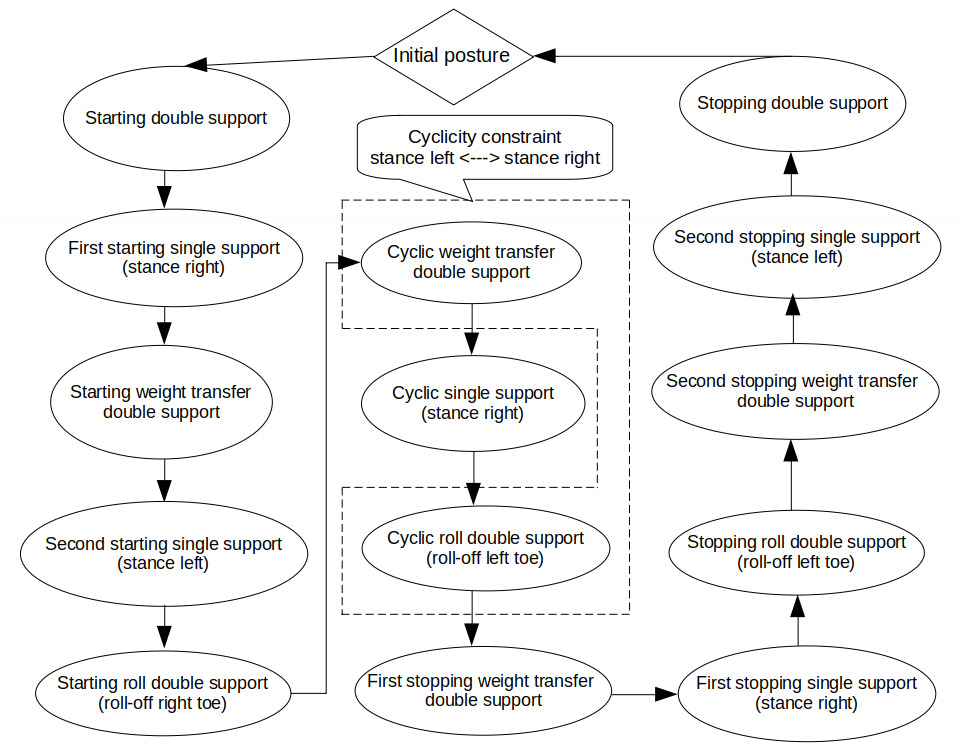

Supplement: Supplementary file 2 [file DataSheet1.zip › Data Sheet 1/Supplementary Images/S9-downstairs_schema.jpeg]

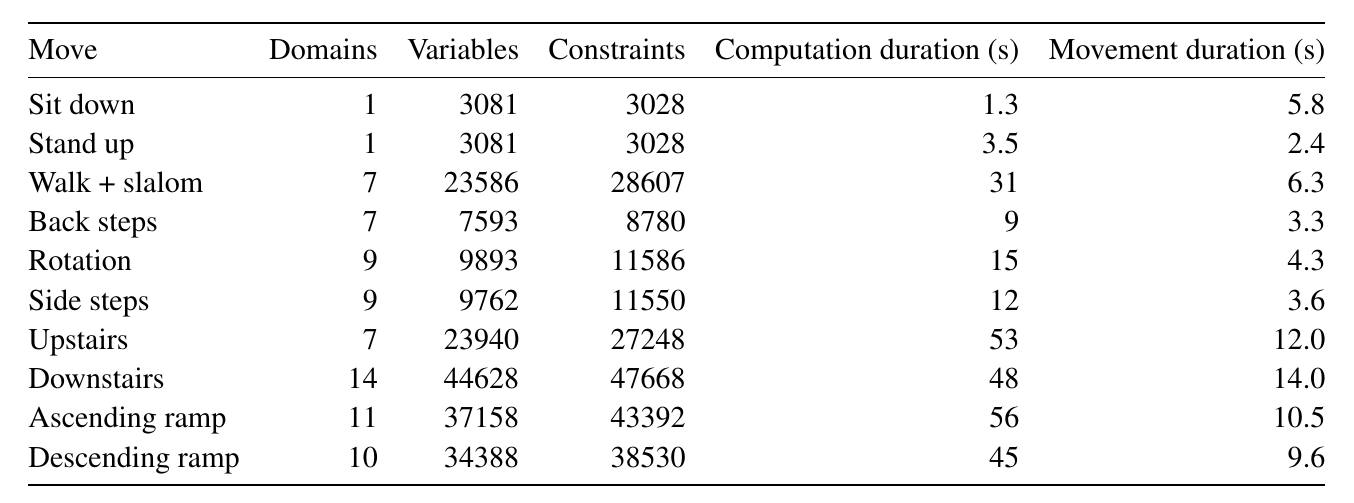

Supplement: Supplementary file 3 [file DataSheet2.zip › tables/Tables S1.png]

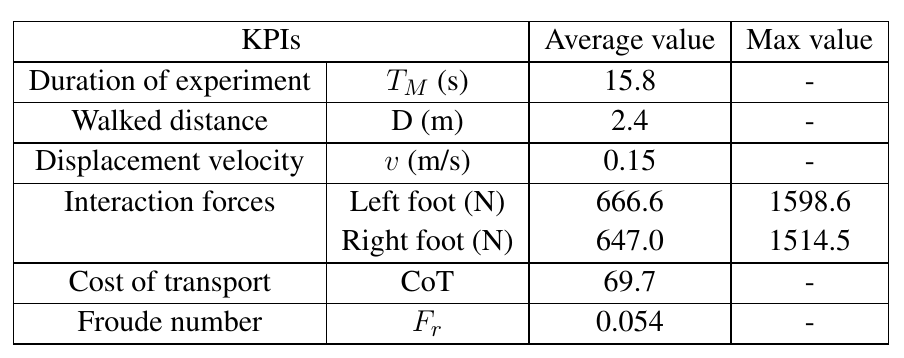

Supplement: Supplementary file 3 [file DataSheet2.zip › tables/Tables S2.png]

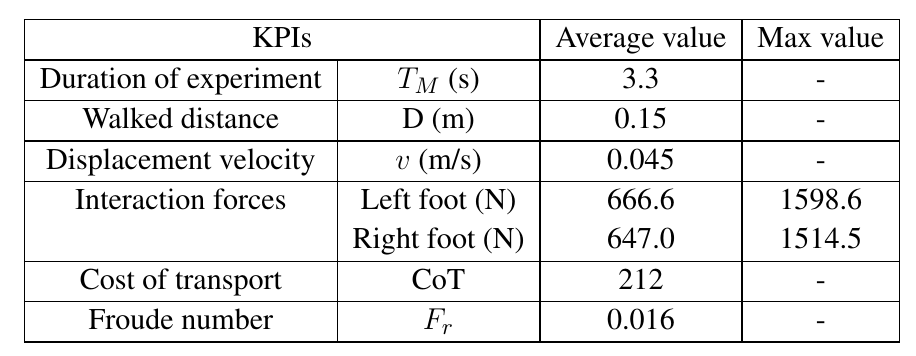

Supplement: Supplementary file 3 [file DataSheet2.zip › tables/Tables S3.png]

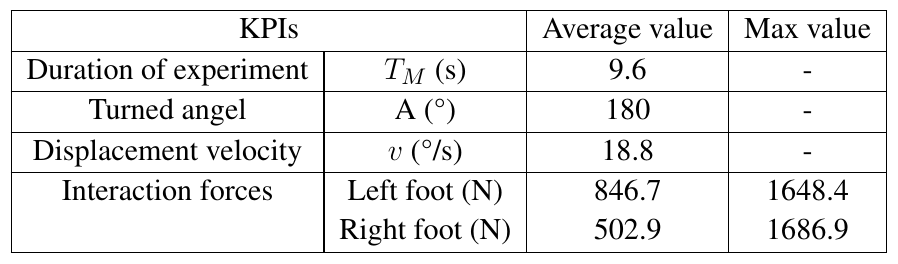

Supplement: Supplementary file 3 [file DataSheet2.zip › tables/Tables S4.png]

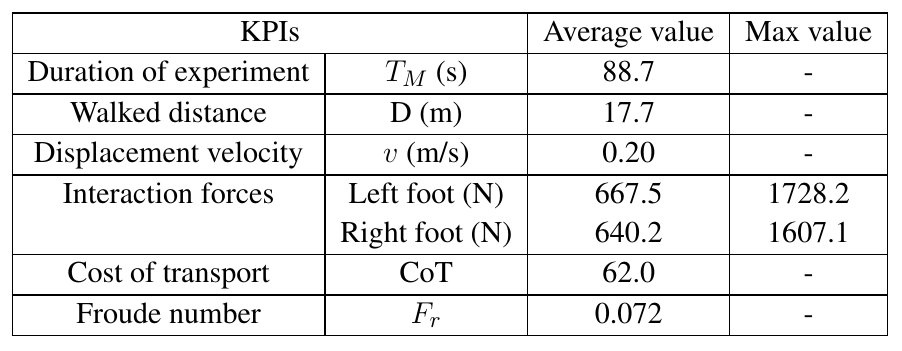

Supplement: Supplementary file 3 [file DataSheet2.zip › tables/Tables S5.png]

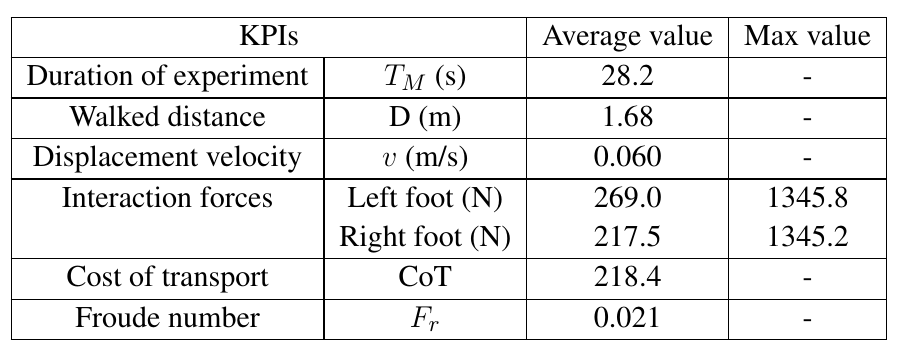

Supplement: Supplementary file 3 [file DataSheet2.zip › tables/Tables S6.png]

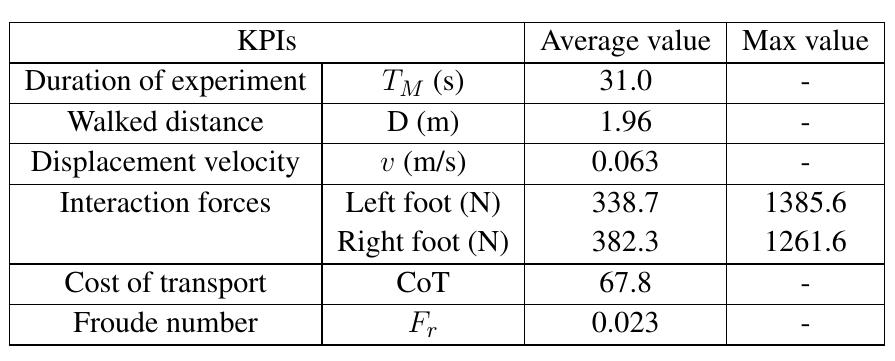

Supplement: Supplementary file 3 [file DataSheet2.zip › tables/Tables S7.png]

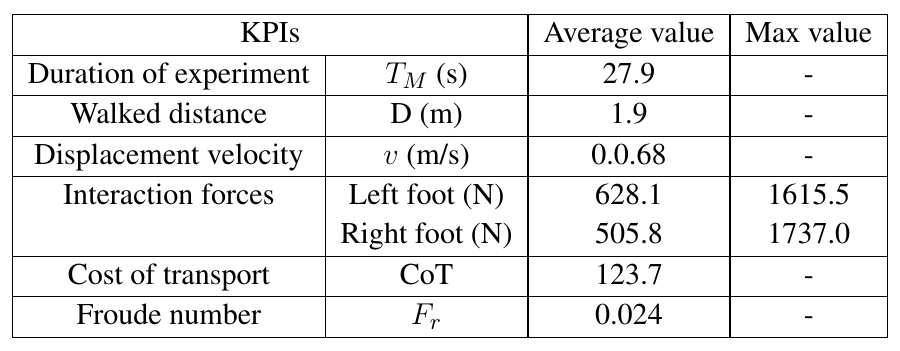

Supplement: Supplementary file 3 [file DataSheet2.zip › tables/Tables S8.png]

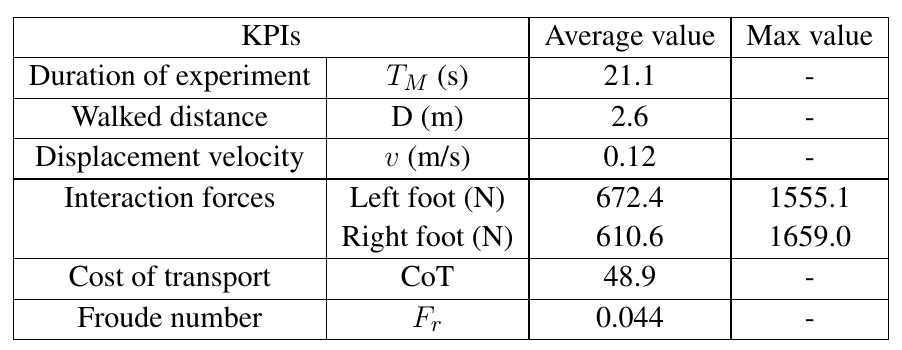

Supplement: Supplementary file 3 [file DataSheet2.zip › tables/Tables S9.png]
